# New Antioxidant Caffeate Esters of Fatty Alcohols Identified in *Robinia pseudoacacia*

**DOI:** 10.3390/molecules29235673

**Published:** 2024-11-30

**Authors:** Ágnes M. Móricz, Márton Baglyas, András Darcsi, József Balla, Gertrud E. Morlock

**Affiliations:** 1 Plant Protection Institute, HUN-REN Centre for Agricultural Research, Fehérvári út 132–144, 1116 Budapest, Hungary; baglyas.marton@atk.hun-ren.hu; 2 Doctoral School, Semmelweis University, Üllői út 26, 1085 Budapest, Hungary; 3 Pharmaceutical Chemistry and Technology Department, National Center for Public Health and Pharmacy, Szabolcs utca 33, 1135 Budapest, Hungary; darcsi.andrew@gmail.com; 4 Department of Inorganic and Analytical Chemistry, Faculty of Chemical Technology and Biotechnology, Budapest University of Technology and Economics, Szent Gellért tér 4, 1111 Budapest, Hungary; balla.jozsef@vbk.bme.hu; 5 Institute of Nutritional Science, Chair of Food Science, Justus Liebig University Giessen, Heinrich-Buff-Ring 26−32, 35392 Giessen, Germany; gertrud.morlock@uni-giessen.de

**Keywords:** black locust (*Robinia pseudoacacia* L.), phenolic esters of fatty alcohols, high-performance thin-layer chromatography—effect-directed analysis (HPTLC–EDA), antioxidant assay, heated electrospray high-resolution mass spectrometry (HESI-HRMS), bioassay-guided isolation, solid-phase extraction (SPE), reversed-phase high-performance liquid chromatography with diode array detection (RP-HPLC–DAD), nuclear magnetic resonance (NMR) spectroscopy, attenuated total reflectance Fourier-transform infrared (ATR-FTIR) spectroscopy, gas chromatography-mass spectrometry (GC–MS)

## Abstract

The stem bark of black locust (*Robinia pseudoacacia* L.) was extracted, and nine antioxidant compounds (**R1**–**R9**) were detected by high-performance thin-layer chromatography combined with the radical scavenging 2,2-diphenyl-1-picrylhydrazyl (DPPH•) assay, multi-detection, and heated electrospray high-resolution mass spectrometry. For structure elucidation, the methanolic crude extract was fractionated by solid-phase extraction, and the compounds were isolated by reversed-phase high-performance liquid chromatography with diode array detection. The structures of isolated compounds were elucidated by nuclear magnetic resonance and attenuated total reflectance Fourier-transform infrared spectroscopy as well as gas chromatography-mass spectrometry to determine the double bond position. 3-*O*-Caffeoyl oleanolic acid (**R1**), oleyl (**R2**), octadecyl (**R3**), gadoleyl (**R4**), eicosanyl (**R5**), (*Z*)-9-docosenyl (**R6**), docosyl (**R7**), tetracosyl (**R8**), and hexacosanyl (**R9**) caffeates were identified. While **R1** has been reported in *R. pseudoacacia* stem bark, the known **R3**, **R5**, **R7**, **R8**, and **R9** are described for the first time in this species, and the **R2**, **R4**, and **R6** are new natural compounds. All nine caffeates demonstrated antioxidant activity. The antioxidant effects of the isolated compounds **R1**–**R8** were quantified by a microplate DPPH• assay, with values ranging from 0.29 to 1.20 mol of caffeic acid equivalents per mole of isolate.

## 1. Introduction

The North American black locust (*Robinia pseudoacacia* L., family Fabaceae) has been widely planted all over the world, initially as an ornamental tree and later for soil and water conversation, like in Europe since the 17th century [1,2]. It is a fast-growing tree that reproduces both sexually and vegetatively; therefore, it has become one of the most aggressively invasive woody plants with a high biomass worldwide [2]. Due to its allelopathic potential, it often overgrows the indigenous plants, and as a nitrogen-fixing species, it can alter the native vegetation [3]. Despite its environmental drawbacks, it offers economic benefits, particularly in the honey and wood industries [4]. Furthermore, black locust has been used in traditional folk medicine, especially in Europe and Asia, due to its astringent, cholagogue, diuretic, anti-inflammatory, purgative, spasmolytic, and sedative properties, as well as Cherokee treated toothache with it [5,6]. Its beneficial effects [7,8], such as antibacterial, antifungal, antioxidant, anti-inflammatory, and cytotoxic properties, are primarily attributed to the high content of diverse phenoloids [9]. Flowers and leaves are rich sources of phenolic acids (e.g., caffeoylquinic acids, caffeic and coumaric acids and their hexosides, coumaroylquinic acids, ellagic acid hexoside, gallic acid, and *p*-hydroxybenzoic acid), flavonoids (apigenin, catechin, procyanidin dimers and trimers, quercetin and kaempferol derivatives, and vescalagins), and tannins [9,10], all of which exhibit various biological activities. The diversity of flavonoid aglycones and hydroxycinnamic acid derivatives originated from propolis and nectar-derived kaempferol glycosides enables the floral authentication of black locust honey [11]. However, black locust also contains toxic glycoproteins, lectins, and the homo-monoterpene robinlin that can possess pharmacological activities beyond cytotoxicity [12].

The wood of *R. pseudoacacia* is predominantly composed of structural polysaccharides (e.g., cellulose, hemicellulose, and lignin) that conceal a wide range of valuable compounds [13], and it can be the source of biofuels [14]. The heartwood contains several phenolic acids (e.g., caffeic acid, chlorogenic acid, ellagic acid, ferulic acid, *p*-coumaric acid, gallic acid, ellagic acid, *p*-hydroxybenzoic acid, and protocatechuic acid), flavonoids (e.g., di-*O*-methylquercetin B, quercetin, epigallocatechin, fustin, catechin, kaempferol, myricetin, procyanidin dimer, robinetin, and dihydrorobinetin), along with stilbenes (resveratrol and piceatannol) [15,16,17]. Interestingly, the bark lacks robinetin, but dihydrorobinetin and phenolic acids like catechin, epicatechin, caffeic acid, and ferulic acid have been detected as defensive compounds in the bark [18,19].

Plant phenolics play a pivotal role in plant growth, development, and defense by displaying antioxidant, antimicrobial, allelopathic, and UV-blocking effects [20]. Reactive oxygen species (ROS) and free radicals are essential for cell signaling and other vital physiological processes. However, during various diseases, including inflammatory and infectious conditions, their overproduction can lead to potential cellular damage [20,21,22]. Natural antioxidants, such as phenolic compounds, can diminish this unfavorable effect by scavenging the free radicals and converting them into stable forms [23]. High-performance thin-layer chromatography combined with multi-detection (HPTLC–UV/VIS/FLD), the 2,2-diphenyl-1-picrylhydrazyl (DPPH•) assay, and derivatization via the Natural Product reagent A ensure an efficient, high-throughput screening for identifying antioxidant compounds in complex matrices, such as plant extracts [24,25,26]. It is an effective monitoring tool in bioactivity-guided compound isolation [27].

The study aimed at screening, characterization, isolation, and identification of antioxidant compounds from the methanolic bark extracts of the black locust. Various analytical techniques were utilized, including reversed-phase (RP)-HPTLC–DPPH• assay, RP-HPTLC–UV/VIS/FLD–densitometry, RP-HPTLC–heated electrospray high-resolution mass spectrometry (HESI-HRMS), reversed-phase high-performance liquid chromatography diode array detection (RP-HPLC–DAD), attenuated total reflectance Fourier-transform infrared (ATR–FTIR) spectroscopy, nuclear magnetic resonance (NMR) spectroscopy, and gas chromatography–mass spectrometry (GC–MS). The antioxidant activity of the isolated compounds was assessed by a DPPH• microplate assay.

## 2. Results and Discussion

### 2.1. RP-HPTLC–DPPH• Assay Screening and Assignment by RP-HPTLC–HESI-HRMS

**Figure 1:**
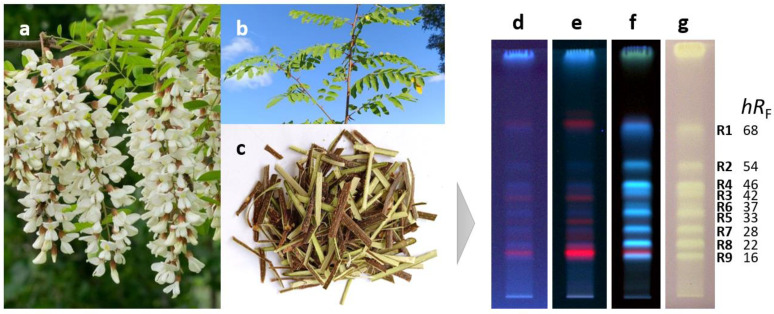
Flowers (**a**), stem with leaves (**b**), and stem bark (**c**) of *Robinia pseudoacacia* along with HPTLC chromatograms of bark crude extract (3 µL) separated on RP18 plates with acetonitrile-ethanol 3:2 *V*/*V* and detected at 254 nm (**d**), 365 nm (**e**), and after derivatization with natural product reagent A at 365 nm (**f**) as well as after the DPPH• assay under white light illumination (**g**) revealing the antioxidant compounds **R1**–**R9**.

Antioxidant compounds of the methanolic crude extract obtained from the black locust bark were separated on RP18 HPTLC plates using acetonitrile—ethanol 3:2 *V*/*V* as a mobile phase and detected via fluorescence detection (FLD) after derivatization with the Natural Product reagent A and via white light illumination (Vis) after the radical scavenging DPPH• assay (Figure 1d–g). Nine antioxidant compound zones at *hR*_F_ 16 (**R9**), 22 (**R8**), 28 (**R7**), 33 (**R5**), 37 (**R6**), 42 (**R3**), 46 (**R4**), 54 (**R2**), and 68 (**R1**) were revealed. Via the derivatization with the Natural Product reagent A, the natively weak blue fluorescence of the zones **R1**–**R9** was enhanced, indicating that the compounds responsible for the antioxidant effect belong to the group of phenolics.

This hypothesis (regarding the presence of phenolics) was confirmed by their densitometrically recorded RP-HPTLC–UV spectra showing characteristic absorption bands between 300 and 350 nm (Figure S1). The antioxidant compounds were further characterized by RP-HPTLC–HESI-HRMS. In the positive ionization mode, the intensity of signals corresponding to sodium adducts ([M+Na]^+^) was low, whereas in the negative ionization mode, signals of deprotonated molecules ([M−H]^−^) were intense (Table 1).

### 2.2. Fractionation by Solid-Phase Extraction and Isolation by RP-HPLC–DAD

**Figure 2:**
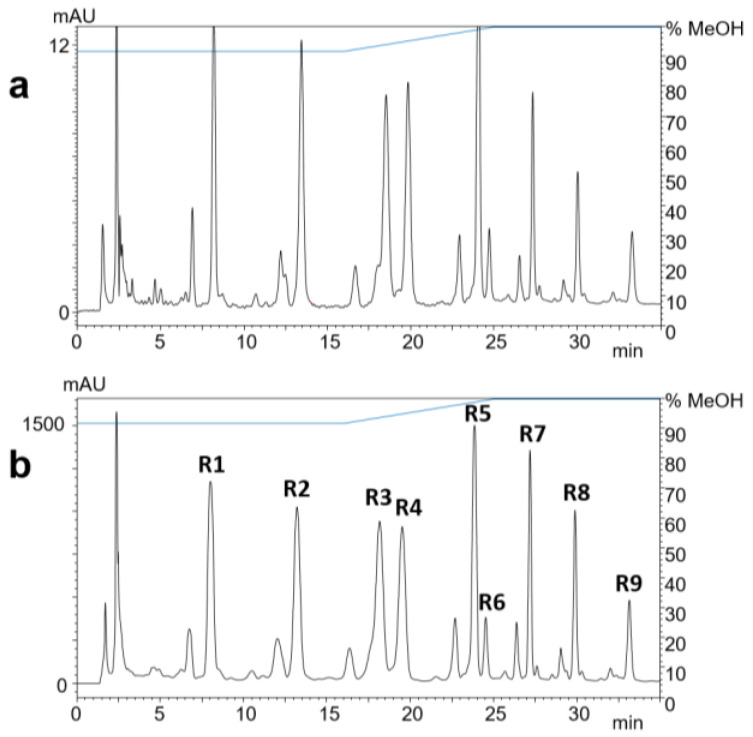
RP-HPLC-UV chromatograms at 323 nm of 1 µL (**a**) and 100 µL (**b**) of black locust bark extract after SPE. Compounds **R1**–**R9** were identified by ESI-MS.

The methanolic crude extract was fractionated by reversed-phase solid-phase extraction (SPE). The separation and peak identification of the compounds were achieved by RP-HPLC–DAD–ESI-MS. The isolation from the ethanol eluate was carried out by RP-HPLC–DAD (Figure 2). The yields of compounds **R1**–**R9** ranged from 0.9 to 2.3 mg (Table 1), which were used for subsequent structure elucidation.

### 2.3. Results of NMR and ATR-FTIR Spectra Recording

The NMR (Figures S3–S35) and ATR-FTIR spectra (Figures S36–S41) were recorded, and the data were compiled and listed as follows for 3-*O*-caffeoyl oleanolic acid (**R1**): IR (ATR) *ν*_max_ 3191, 2941, 2927, 2854, 1696, 1600, 1524, 1463, 1389, 1365, 1266, 1170, 1146, 1117, 1019 cm^–1^; ^1^H and ^13^C NMR data (Table 2).

Oleyl caffeate (**R2**): IR (ATR) *ν*_max_ 2924, 2854, 1712, 1593, 1509, 1460, 1370, 1263, 1167, 1121, 1091, 1050, 1018 cm^–1^; ^1^H NMR (CD_3_OD, 600 MHz) *δ* 7.53 (d, *J* = 16.0 Hz, 1H, H-7′), 7.04 (d, *J* = 2.1 Hz, 1H, H-1′), 6.94 (dd, *J* = 8.3, 2.1 Hz, 1H, H-5′), 6.78 (d, *J* = 8.1 Hz, 1H, H-4′), 6.25 (d, *J* = 15.9 Hz, 1H, H-8′), 5.34 (m, 2H, H-9, H-10), 4.17 (t, *J* = 6.6 Hz, 2H, H-1), 2.03 (m, 4H, H-8, H-11), 1.69 (p, *J* = 7.0 Hz, 2H, H-2), 1.41 (m, 2H, H-3), 1.29 (br s, 20H, H-4–H-7, H-12–H-17), 0.91 (m, 3H, H-18); ^13^C NMR (CD_3_OD, 151 MHz) *δ* (2D HSQC, HMBC) 169.5 (C, C-9′), 149.7 (C, C-3′), 146.9 (C, C-2′), 146.8 (CH, C-7′), 130.8 (CH, C-9), 130.8 (CH, C-10), 127.8 (C, C-6′), 122.9 (CH, C-5′), 116.6 (CH, C-4′), 115.3 (CH, C-8′), 115.1 (CH, C-1′), 65.6 (CH_2_, C-1), 33.2 (CH_2_, C-16), 30.9–30.2 (CH_2_, C-4–C-7, C-12–C-15), 29.8 (CH_2_, C-2), 28.0 (CH_2_, C-8, C-11), 27.1 (CH_2_, C-3), 23.4 (CH_2_, C-17), 14.4 (CH_3_, C-18)
Octadecyl caffeate (**R3**): IR (ATR) *ν*_max_ 3222, 2918, 2851, 1710, 1593, 1520, 1466, 1382, 1269, 1165, 1119, 1077, 1049 cm^–1^; ^1^H NMR (CD_3_OD, 600 MHz) *δ* 7.53 (d, *J* = 15.9 Hz, 1H, H-7′), 7.04 (d, *J* = 2.1 Hz, 1H, H-1′), 6.94 (dd, *J* = 8.1, 2.1 Hz, 1H, H-5′), 6.78 (d, *J* = 8.2 Hz, 1H, H-4′), 6.25 (d, *J* = 15.9 Hz, 1H, H-8′), 4.17 (t, *J* = 6.6 Hz, 2H, H-1), 1.70 (p, *J* = 6.8 Hz, 2H, H-2), 1.41 (m, 2H, H-3), 1.29 (br s, 28H, H-4–H-17), 0.90 (t, *J* = 7.0 Hz, 3H, H-18); ^13^C NMR (CD_3_OD, 151 MHz) *δ* 169.4 (C, C-9′), 149.6 (C, C-3′), 146.8 (C, C-2′), 146.8 (CH, C-7′), 127.7 (C, C-6′), 122.9 (CH, C-5′), 116.5 (CH, C-4′), 115.2 (CH, C-8′), 115.1 (CH, C-1′), 65.6 (CH_2_, C-1), 33.1 (CH_2_, C-16), 30.8–30.6 (CH_2_, C-6–C-15), 30.5 (CH_2_, C-4), 30.3 (CH_2_, C-5), 29.8 (CH_2_, C-2), 27.1 (CH_2_, C-3), 23.7 (CH_2_, C-17), 14.4 (CH_3_, C-18)
Gadoleyl caffeate (**R4**): IR (ATR) *ν*_max_ 2925, 2854, 1713, 1683, 1648, 1592, 1540, 1459, 1347, 1266, 1165, 1122, 1050, 1013 cm^–1^; ^1^H NMR (CD_3_OD, 600 MHz) *δ* 7.53 (d, *J* = 16.0 Hz, 1H, H-7′), 7.04 (d, *J* = 2.1 Hz, 1H, H-1′), 6.94 (dd, *J* = 8.1, 2.0 Hz, 1H, H-5′), 6.78 (d, *J* = 8.1 Hz, 1H, H-4′), 6.25 (d, *J* = 15.9 Hz, 1H, H-8′), 5.34 (m, 2H, H-9, H-10), 4.17 (t, *J* = 6.6 Hz, 2H, H-1), 2.03 (m, 4H, H-8, H-11), 1.69 (p, *J* = 7.1 Hz, 2H, H-2), 1.41 (m, 2H, H-3), 1.29 (br s, 24H, H-4–H-7, H-12–H-19), 0.91 (m, 3H, H-20); ^13^C NMR (CD_3_OD, 151 MHz) *δ* (2D HSQC, HMBC) 169.5 (C, C-9′), 149.6 (C, C-3′), 146.9 (C, C-2′), 146.8 (CH, C-7′), 130.8 (CH, C-9), 130.8 (CH, C-10), 127.8 (C, C-6′), 123.0 (CH, C-5′), 116.6 (CH, C-4′), 115.3 (CH, C-8′), 115.1 (CH, C-1′), 65.6 (CH_2_, C-1), 33.1 (CH_2_, C-18), 30.9–30.2 (CH_2_, C-4–C-7, C-12–C-17), 29.8 (CH_2_, C-2), 28.0 (CH_2_, C-8, C-11), 27.1 (CH_2_, C-3), 23.4 (CH_2_, C-19), 14.4 (CH_3_, C-20)
Eicosanyl caffeate (**R5**): IR (ATR) *ν*_max_ 3327, 2918, 2851, 1713, 1599, 1523, 1468, 1380, 1265, 1165, 1118, 1089, 1048 cm^–1^; ^1^H NMR (CD_3_OD, 600 MHz) *δ* 7.53 (d, *J* = 15.9 Hz, 1H, H-7′), 7.04 (d, *J* = 2.0 Hz, 1H, H-1′), 6.94 (dd, *J* = 8.3, 2.1 Hz, 1H, H-5′), 6.78 (d, *J* = 8.2 Hz, 1H, H-4′), 6.25 (d, *J* = 15.9 Hz, 1H, H-8′), 4.17 (t, *J* = 6.6 Hz, 2H, H-1), 1.70 (p, *J* = 6.9 Hz, 2H, H-2), 1.41 (m, 2H, H-3), 1.29 (br s, 32H, H-4–H-19), 0.90 (t, *J* = 7.0 Hz, 3H, H-20); ^13^C NMR (CD_3_OD, 151 MHz) *δ* (2D HSQC, HMBC) 169.5 (C, C-9′), 149.7 (C, C-3′), 146.9 (C, C-2′), 146.8 (CH, C-7′), 127.8 (C, C-6′), 122.9 (CH, C-5′), 116.5 (CH, C-4′), 115.2 (CH, C-8′), 115.1 (CH, C-1′), 65.6 (CH_2_, C-1), 33.1 (CH_2_, C-18), 30.8–30.0 (CH_2_, C-4–C-17), 29.8 (CH_2_, C-2), 27.1 (CH_2_, C-3), 23.7 (CH_2_, C-19), 14.4 (CH_3_, C-20)
(*Z*)-9-docosenyl caffeate (**R6**): ^1^H NMR (CD_3_OD, 600 MHz) *δ* 7.53 (d, *J* = 16.0 Hz, 1H, H-7′), 7.04 (d, *J* = 2.0 Hz, 1H, H-1′), 6.94 (dd, *J* = 8.0, 1.7 Hz, 1H, H-5′), 6.78 (d, *J* = 8.1 Hz, 1H, H-4′), 6.25 (d, *J* = 15.9 Hz, 1H, H-8′), 5.34 (m, 2H, H-9, H-10), 4.17 (t, *J* = 6.5 Hz, 2H, H-1), 2.03 (m, 4H, H-8, H-11), 1.70 (m, 2H, H-2), 1.41 (m, 2H, H-3), 1.29 (br s, 28H, H-4–H-7, H-12–H-21), 0.90 (t, *J* = 6.7 Hz, 3H, H-22)
Docosyl caffeate (**R7**): IR (ATR) *ν*_max_ 2917, 2850, 1716, 1583, 1512, 1467, 1433, 1373, 1259, 1168, 1120, 1056 cm^–1^; ^1^H NMR (CD_3_OD, 600 MHz) *δ* 7.53 (d, *J* = 15.9 Hz, 1H, H-7′), 7.04 (d, *J* = 2.1 Hz, 1H, H-1′), 6.94 (dd, *J* = 8.2, 2.1 Hz, 1H, H-5′), 6.78 (d, *J* = 8.2 Hz, 1H, H-4′), 6.25 (d, *J* = 15.8 Hz, 1H, H-8′), 4.17 (t, *J* = 6.6 Hz, 2H, H-1), 1.70 (p, *J* = 6.8 Hz, 2H, H-2), 1.40 (m, 2H, H-3), 1.29 (br s, 36H, H-4–H-21), 0.90 (t, *J* = 7.1 Hz, 3H, H-22); ^13^C NMR (CD_3_OD, 151 MHz) *δ* (2D HSQC, HMBC) 169.5 (C, C-9′), 149.7 (C, C-3′), 146.8 (C, C-2′), 146.6 (CH, C-7′), 127.8 (C, C-6′), 122.9 (CH, C-5′), 116.6 (CH, C-4′), 115.3 (CH, C-8′), 115.1 (CH, C-1′), 65.6 (CH_2_, C-1), 33.2 (CH_2_, C-20), 30.9–30.0 (CH_2_, C-4–C-19), 29.9 (CH_2_, C-2), 27.1 (CH_2_, C-3), 23.7 (CH_2_, C-21), 14.4 (CH_3_, C-22)
Tetracosyl caffeate (**R8**): ^1^H NMR (CD_3_OD, 600 MHz) *δ* 7.53 (d, *J* = 15.8 Hz, 1H, H-7′), 7.04 (d, *J* = 2.1 Hz, 1H, H-1′), 6.94 (dd, *J* = 8.0, 1.8 Hz, 1H, H-5′), 6.78 (d, *J* = 8.1 Hz, 1H, H-4′), 6.25 (d, *J* = 15.9 Hz, 1H, H-8′), 4.17 (t, *J* = 6.7 Hz, 2H, H-1), 1.70 (p, *J* = 6.9 Hz, 2H, H-2), 1.40 (m, 2H, H-3), 1.29 (br s, 40H, H-4–H-23), 0.90 (t, *J* = 6.8 Hz, 3H, H-24)
Hexacosanyl caffeate (**R9**): ^1^H NMR (CD_3_OD, 600 MHz) for *O*-caffeoyl moiety *δ* 7.53 (d, *J* = 15.7 Hz, 1H, H-7′), 7.04 (d, *J* = 2.1 Hz, 1H, H-1′), 6.94 (dd, *J* = 8.0, 1.8 Hz, 1H, H-5′), 6.78 (d, *J* = 8.2 Hz, 1H, H-4′), 6.25 (d, *J* = 15.8 Hz, 1H, H-8′)

### 2.4. Structure Elucidation of the Isolated Compounds

The NMR and ATR-FTIR spectroscopy data were consistent with HESI-HRMS data and in good agreement with the literature cited. The downfield region of the ^1^H NMR spectra of the isolated compounds **R1**–**R9** highly resembled each other with a set of resonances at *δ*_H_ 7.04–7.03 (d, *J* ≈ 2 Hz, 1H, H-1′), 6.94 (dd, *J* ≈ 8 and 2 Hz, 1H, H-5′), and 6.78 (d, *J* ≈ 8 Hz, 1H, H-4′) ppm suggesting the presence of a 1,2,4-trisubstituted aromatic ring. Moreover, ^1^H signals at *δ*_H_ 7.53–7.52 (d, *J* ≈ 16 Hz, 1H, H-7′) and 6.25–6.24 (d, *J* ≈ 16 Hz, 1H, H-8′) ppm implied a *trans*-oriented, disubstituted carbon-carbon double bond. The ^13^C or the ^1^H–^13^C HMBC spectra of compounds **R1**–**R8** revealed one ester carbonyl carbon at *δ*_C_ 169.5–169.2 located at the C-9′ position, as evidenced by HMBC correlations H-7′/C-9′ and H-8′/C-9′. Besides, they exhibited typical carbon signals at *δ*_C_ 149.7–149.6 (C, C-3′), 146.9–146.7 (C, C-2′), 146.8–146.6 (CH, C-7′), 127.8–127.7 (C, C-6′), 123.0–122.9 (CH, C-5′), 116.6–116.5 (CH, C-4′), 115.6–115.2 (CH, C-8′), and 115.1 (CH, C-1′) ppm, indicating that their structure contains an *O*-caffeoyl basic skeleton [28,29], which were corroborated by the ATR-FTIR absorption bands at around 3350–3200 cm^–1^ (O–H stretch) and at 1716–1710 cm^–1^ (*α*,*β*-unsaturated ester C=O stretch). The connectivity between the aromatic ring and the double bond was confirmed by long-range HMBC correlations H-8′/C-6′, H-7′/C-1′, H-7′/C-5′, and H-7′/C-6′. The ^13^C chemical shifts could not be determined for compounds **R6** and **R8** due to their low isolated quantity of below 1 mg (Table 1).

**Figure 3:**
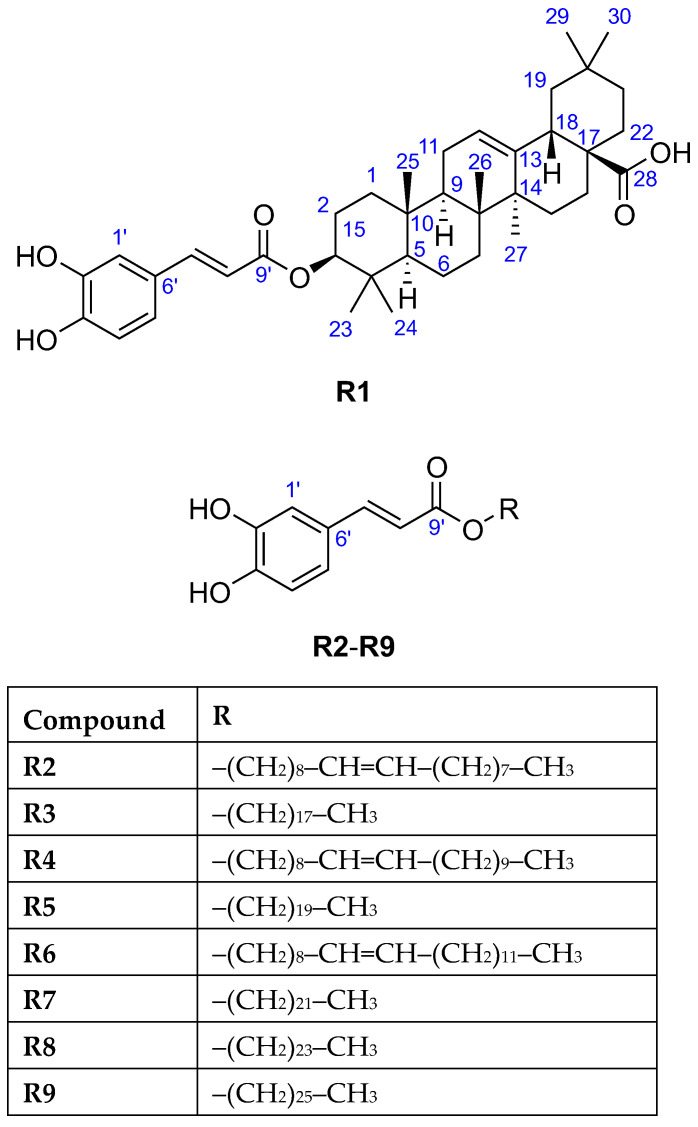
The chemical structures of the isolated compounds **R1**–**R9**.

The HESI-HRMS spectrum of **R1** showed a deprotonated molecule peak at *m*/*z* 617.3848 [M−H]^−^ establishing its molecular formula as C_39_H_54_O_6_ (Table 1), which corresponded to 13 double bond equivalents. Its ^1^H NMR spectrum (Table 2) indicated the presence of seven isolated methyl groups at *δ*_H_ 1.19 (s, 3H, H-27), 1.01 (s, 3H, H-25), 0.97 (s, 3H, H-24), 0.95 (s, 3H, H-30), 0.91 (s, 3H, H-23), 0.91 (s, 3H, H-29), and 0.84 (s, 3H, H-26) ppm, an olefinic proton at *δ*_H_ 5.26 (t, *J* = 3.6 Hz, 1H, H-12) ppm, and an oxymethine proton at *δ*_H_ 4.57 (dd, *J* = 11.5, 4.3 Hz, 1H, H-3) ppm. The ^13^C NMR spectrum (Table 2) revealed 30 carbon resonances excluding the *O*-caffeoyl moiety, including seven methyl carbons at *δ*_C_ 33.6 (C-29), 28.7 (C-23), 26.4 (C-27), 24.0 (C-30), 17.7 (C-26), 17.3 (C-24), 15.9 (C-25) ppm, two olefinic carbons at *δ*_C_ 145.3 (C-13), 123.5 (C-12) ppm, one oxygenated carbon at *δ*_C_ 82.3 (C-3) ppm, as well as one carboxylic carbon at *δ*_C_ 182.1 (C-28) ppm. The caffeate and carboxylic moiety and the carbon-carbon double bond account for eight double bond equivalents, implying a pentacyclic triterpene skeleton with seven angular methyl groups. Based on the spectral data along with the 2D homo- and heteronuclear correlations and by comparing the ^1^H, ^13^C NMR, and ATR-FTIR data with those reported in the literature [30,31], the triterpene was identified as oleanolic acid. The point of attachment between the *O*-caffeoyl moiety and oleanolic acid was determined by the downfield chemical shifts of H-3 at *δ*_H_ 4.57 ppm and C-3 at *δ*_C_ 82.3 ppm, and a key HMBC correlation was observed from H-3 to C-9′, supporting the substitution of oleanolic acid at the C-3 position. In addition, the large coupling constant between H-2_ax_ and H-3 (^3^*J*_H-2ax–H-3_ = 11.5 Hz) confirmed that H-3 occupied an *α*-axial position, thereby indicating that the *O*-caffeoyl moiety was *β*-oriented. Thus, compound **R1** was elucidated as 3-*O*-caffeoyl oleanolic acid (Figure 3).

The ^1^H NMR spectra of the four isolated compounds **R3**, **R5**, **R7**, and **R8** were remarkably similar, which was consistent with literature [32,33,34,35,36], displaying another set of signals at *δ*_H_ 4.17 (t, *J* ≈ 6.5 Hz, 2H, H-1), 1.70 (p, *J* ≈ 7 Hz, 2H, H-2), 1.41 (m, 2H, H-3), 1.29 (br s, varied integrals), 0.90 (t, *J* ≈ 7 Hz, 3H, terminal CH_3_) ppm suggestive for the presence of a saturated fatty acid moiety in their structures. It was verified by the observed ^13^C signals at *δ*_C_ 65.6 (CH_2_, C-1), 33.2–33.1 (CH_2_, C-16(**R3**)/C-18(**R5**)/C-20(**R7**)), 30.9–30.0 (CH_2_, C-4–C-15(**R3**)/C-17(**R5**)/C-19(**R7**)), 29.9–29.8 (CH_2_, C-2), 27.1 (CH_2_, C-3), 23.7 (CH_2_, C-17(**R3**)/C-19(**R5**)/C-21(**R7**)), 14.4 (CH_3_, C-18(**R3**)/C-20(**R5**)/C-22(**R7**)) ppm [28,37]. In the HESI-HRMS spectra, deprotonated molecules were observed at *m*/*z* 431.3162 [M−H]^−^ (**R3**), *m*/*z* 459.3475 [M−H]^−^ (**R5**), *m*/*z* 487.3788 [M−H]^−^ (**R7**), and *m*/*z* 515.4101 [M−H]^−^ (**R8**), corresponding to the molecular formulae C_27_H_44_O_4_ (**R3**), C_29_H_48_O_4_ (**R5**), C_31_H_52_O_4_ (**R7**), and C_33_H_56_O_4_ (**R8**) (Table 1) that suspected a long-chain series with a two methylene group difference between adjacent members. The chemical formula of the aliphatic chains could be determined based on the fact that **R3**, **R5**, **R7**, and **R8** were caffeate esters: C_18_H_37_, C_20_H_41_, C_22_H_45_, and C_24_H_49_, respectively. Thus, the isolates were identified as octadecyl caffeate (**R3**), eicosanyl caffeate (**R5**), docosyl caffeate (**R7**), and tetracosyl caffeate (**R8**), respectively (Figure 3).

Due to the low purity and isolated amount of compound **R9**, only limited structural information could be inferred from its ^1^H NMR spectrum revealing the characteristic signals of *O*-caffeoyl moiety at *δ*_H_ 7.53 (d, *J* = 15.7 Hz, 1H, H-7′), 7.04 (d, *J* = 1.8 Hz, 1H, H-1′), 6.94 (dd, *J* = 7.8 and 1.9 Hz, 1H, H-5′), 6.78 (d, *J* = 8.2 Hz, 1H, H-4′), and 6.25 (d, *J* = 15.8 Hz, 1H, H-8′) ppm. The HESI-HRMS spectrum displayed a deprotonated molecule peak at *m*/*z* 543.4419 [M−H]^−^ indicating the molecular formula as C_35_H_60_O_4_ (Table 1). Being a caffeate ester, the chemical formula C_26_H_53_ was deduced for the fatty alcohol moiety, thus compound **R9** was assigned as hexacosanyl caffeate (Figure 3) [38].

### 2.5. GC–MS for Assignment of the Double Bond Position

Based on the HESI-HRMS analyses (Table 1), deprotonated molecules were detected at *m*/*z* 429.3005 [M−H]^−^ (**R2**), 457.3318 [M−H]^−^ (**R4**), and *m*/*z* 485.3631 [M−H]^−^ (**R6**), corresponding to the molecular formulae C_27_H_42_O_4_ (**R2**), C_29_H_46_O_4_ (**R4**), and C_31_H_50_O_4_ (**R6**), indicative of one degree of unsaturation in the fatty alcohol moiety compared to **R3**, **R5**, **R7**, and **R8**. The ^1^H NMR spectra of compounds **R2**, **R4**, and **R6** were similar to each other and that of **R3**, **R5**, **R7**, and **R8**, with the additional resonances at *δ*_H_ 5.34 (m, 2H) and 2.03 (m, 4H) consistent with one carbon-carbon double bond. The presence of a monounsaturated long chain was supported by additional ^13^C signals at *δ*_C_ 130.7–130.5 (CH) and 27.9–27.7 (CH_2_) ppm, indicating the chemical formulae C_18_H_35_ (**R2**), C_20_H_39_ (**R4**), and C_22_H_43_ (**R6**) for aliphatic chains. However, the position of the double bond in the side chain could not be elucidated by NMR spectroscopy; therefore, GC–MS analyses were conducted. Compounds **R2** and **R4** were identified as oleyl and gadoleyl caffeate, respectively, as their aliphatic chains (oleyl and gadoleyl alcohol) were recognized by the NIST mass spectral library search, showing an excellent agreement between the experimental and theoretical EI-MS spectra (Figures S42 and S43). However, the long fatty alcohol chain of compound **R6** does not have a mass spectrum in the databases (Figure S44); therefore, we used its chromatographic retention property for its identification. The measured retention times for **R2**, **R4**, and **R6** were 7.5, 8.4, and 9.3 min, respectively (Figure S45). The identical position and configuration of the double bond (9*Z*) in the fatty alcohol chain of **R6** were confirmed by the linear relationship between the number of carbon atoms and logarithmic retention times (R^2^ = 0.998) (Figure S46), indicating that **R2**, **R4**, and **R6** belong to the same homologous series. Thus, compound **R6** was determined as (*Z*)-9-docosenyl caffeate.

### 2.6. Equivalency Calculation of the Antioxidant Activity of the Isolates by DPPH• Microplate Assay

All isolated compounds exhibited antioxidant effects using the DPPH• microplate assay (Table 3), which confirmed the initial screening results obtained from the RP-HPTLC–DPPH• assay and the assignment by RP-HPTLC–HESI-HRMS. Antioxidant activity of **R1**–**R8** was compared to that of caffeic acid and found to be 0.10–0.35 mg caffeic acid equivalents per mg isolate, corresponding to 0.29–1.20 mol caffeic acid equivalents per mol isolate. The **R1** displayed the strongest antioxidant activity, surpassing caffeic acid at its molar level (1.20 mol caffeic acid equivalent/mol **R1**), while the **R8** demonstrated the weakest activity (0.29 mol caffeic acid equivalent/mol **R8**). The antioxidant effect of **R9** was detected but not quantified due to its low purity.

### 2.7. Progress Achieved in Comparison to Literature

3-*O*-Caffeoyl oleanolic acid (**R1**) has been isolated from different plant organs such as the seeds of *Oenothera biennis* [39], the whole plant of *Leptopus lolonum* [40], the leaves of *Elaeagnus oldhamii* [41], the barks of *Betula platyphylla* var. *japonica* [42], the skins of apples and pears [43], and the stem bark of *R. pseudoacacia* [44]. This compound demonstrated cytotoxic [41,45], antineoplastic [40,42], antibacterial against *Mycobacterium tuberculosis* [46], anti-inflammatory [43,47], anticoronavirus [45], and antioxidant [39] effects.

Phenolic esters with long-chain saturated fatty alcohols (**R3**, **R5**, **R7**, **R8**, and **R9**) were described in various plant species but not from the *Robinia* genus. Among others, the root bark of *Paeonia suffruticosa* [48], leaves of *Artemisia argyi* [49], bark of *Acacia* species [50], and roots of *Ipomoea asarifolia* [51] were reported as a source of octadecyl caffeate (**R3**) that displayed α-glucosidase and α-amylase inhibition [48], antioxidant [36,49], cytotoxic [51], antiproliferative [37], anti-HIV (Human immunodeficiency virus) [52], and anti-inflammatory [37] activities. Eicosanyl caffeate (**R5**) and docosyl caffeate (**R7**) were found in stems of *Wikstroemia scytophylla* [53], roots of *Glycyrrhiza glabra* [33], and *Sophora* species [54,55]. Both exhibited chymotrypsin-like elastase inhibition [33], antiproliferative [37], anti-inflammatory [37], and antioxidant [33,49,56] effects. The isolation of docosyl caffeate (**R7**) from *Thymelaea hirsute* [57], the bark of *Acacia* species [50], its antineoplastic effect [57], and moderate activity against acetyl- and butyrylcholinesterase enzymes [58] have also been reported. Tetracosyl caffeate (**R8**) was described as a constituent of wigs of *Hypericum oblongifolium* [59], the whole plant of *Caragana conferta* [60], roots of *Caesalpinia mimosoides* [61], and bark of *Acacia* species [50], and as a urease inhibitor [62], anti-inflammatory [61], antineoplastic [34], antimicrobial [63], and cytotoxic [61] agent. The stem bark of *Pongamia glabra* [38], bark of *Acacia* species [50], stem bark and leaves of *Inga edulis* [64], and stems of *Hibiscus taiwanensis* [65] were sources of hexacosanyl caffeate (**R9**) that showed antioxidant activity [66]. Synthetic oleyl caffeate **(R2**) exerted inhibitory activity against HIV-1 [67]. To the best of our knowledge, oleyl caffeate **(R2**), gadoleyl caffeate (**R4**), and (*Z*)-9-docosenyl caffeate (**R6**) has not been reported previously as natural product constituents.

Phenolic compounds with hydrogen- or electron-donating properties are potential free radical scavengers that protect biomolecules from oxidative stress. Their antioxidant capacity is structure-related, mainly depending on the number and position of hydroxyl groups attached to the aromatic ring and the presence of sugar or other substituents [68]. Caffeic acid, with its dihydroxylated aromatic ring in *ortho* position, is one of the strongest phenolic antioxidants. Its half-maximal effective concentration (EC_50_) value in the DPPH• assay was similar to that of flavonoid aglycones (quercetin, kaempferol, and epicatechin) and lower than that of the well-known potent antioxidant ascorbic acid or other phenolic acids (e.g., 3-*O*-chlorogenic acid, ferulic acid, *p*-coumaric acid, and *p*-hydroxybenzoic acid) [69,70]. In this study, the antioxidant activity of the isolated compounds was compared to that of caffeic acid, and it was found that 3-*O*-caffeoyl oleanolic acid (**R1**) was stronger, while other isolates were similar or slightly weaker than caffeic acid. These results are in agreement with literature data, as 3-*O*-caffeoyl oleanolic acid (**R1**) exerted lower free radical scavenging activity than ascorbic acid [39], octadecyl caffeate (**R3**) showed an antioxidant effect comparable to caffeic acid and higher than ferulic acid and sinapic acid [36], and hexacosanyl caffeate (**R9**) exhibited a slightly lower activity than caffeic acid [66]. However, in the DPPH• assay, eicosanyl caffeate (**R5**) and docosyl caffeate (**R7**) displayed weaker antioxidant activity (10–15 times higher EC_50_) than gallic acid [33,49], which was found to be a stronger free radical scavenger (two times lower EC_50_) than caffeic acid in the same assay [71].

## 3. Materials and Methods

### 3.1. Materials

HPTLC plates, silica gel 60 RP18, and methanol (MS grade) were purchased from Merck (Darmstadt, Germany). Solvents for extraction and HPTLC (analytical grade) were obtained from Th. Geyer (Renningen, Germany) or Reanal (Budapest, Hungary). The 2,2-diphenyl-1-picrylhydrazyl radical (DPPH•) and caffeic acid (98%) were acquired from Sigma-Aldrich (Steinheim, Germany), and Natural Product reagent A (diphenylboryloxyethylamine or diphenylboric acid *β*-ethylamino ester, 98%) was purchased from Carl Roth (Karlsruhe, Germany). Methanol-*d*_4_ (CD_3_OD, 99.8 atom% D) for NMR measurements was purchased from VWR (Budapest, Hungary), and gradient-grade methanol and acetonitrile for isolation were supplied by Fisher Scientific (Pittsburg, PA, USA).

### 3.2. Sample Origin and Preparation

The stem bark of *R. pseudoacacia* L. was collected in October 2016 in Harta (46°41′45″ N 19°02′26″ E, altitude: 93 m) in the Great Plain of Hungary and dried at room temperature. A voucher sample (PPI-MA-RB1) has been deposited at the herbarium of the Plant Protection Institute, Centre for Agricultural Research, Budapest, Hungary. The dried material was powdered by a coffee grinder (Bosch MKM6000, Stuttgart, Germany) and was extracted with methanol (150 mg/mL) using an ultrasound-assisted extraction for 10 min (Sonorex Super RK 106, Bandelin, Berlin, Germany) and centrifuged for 1 min at 5000× *g* (Dlab D1008, Beijing, China).

### 3.3. High-Performance Thin-Layer Chromatography, Derivatization, and DPPH• Assay

The crude extract (3 µL) was applied onto the RP18 HPTLC plate by the Automatic TLC Sampler 4 (ATS4, CAMAG, Muttenz, Switzerland) as a 7 mm band with an 8 mm distance from the lower edge. HPTLC separation was carried out with a mobile phase of acetonitrile—ethanol 3:2 *V*/*V* in a Twin Trough Chamber (10 cm × 10 cm, CAMAG) up to 80 mm from the lower edge of the plate. The dried chromatogram was detected at 254 nm and 365 nm with the TLC Visualizer (CAMAG), and the UV spectra of selected zones were recorded by a TLC Scanner 4 (CAMAG). To detect phenolics (e.g., flavonoids, anthocyanidines, hydroxyl- and methoxycinnamic acids [72]), the plate was dipped into a 0.5% methanolic solution of Natural Product reagent A, dried, and documented at 365 nm. Free radical scavenging activity was visualized by the HPTLC–DPPH• assay. The chromatogram was immersed into a 0.02% methanolic solution of DPPH•, and the bright zones of antioxidants against a lilac background were documented under white light illumination in the transmittance mode (TLC Visualizer).

### 3.4. HPTLC–HESI-HRMS

For HPTLC–HRMS analysis [73,74], a TLC–MS Interface (CAMAG) or a PlateExpress interface (Advion, Ithaca, NY, USA) equipped with an oval elution head (4 mm × 2 mm) was integrated online between a quaternary pump (Ultimate LPG-3400 XRS, Dionex Softron, Germering, Germany) and a hybrid quadrupole-orbitrap mass spectrometer operated with a heated electrospray ionization probe (HESI-II, Q Exactive Plus, Thermo Fisher Scientific, Bremen, Germany). MS-grade methanol at a flow rate of 0.1 mL/min was used to elute selected zones. The following conditions were applied: spray voltage 3.5 kV, capillary temperature 270 °C, and nitrogen sheath and auxiliary gas with 20 and 10 arbitrary units, respectively, produced by an SF2 compressor (Atlas Copco Kompressoren und Drucklufttechnik, Essen, Germany). HRMS full scan spectra were recorded in both negative and positive ionization modes in the range of *m*/*z* 80–1200 with a resolution of 280,000; the automatic gain control target (AGCT) was set to 3 × 10^6^, and the maximum injection time (IT) was 100 ms. Xcalibur 3.0.63 software (Thermo Fisher Scientific) provided the instrument control and data analysis.

### 3.5. Fractionation by Solid-Phase Extraction

The bark powder (20 g) was extracted three times with 100 mL of methanol by ultrasound-assisted extraction. The combined extracts were filtered (Whatman No. 2 filter paper, Sigma), concentrated by a rotary evaporator to 20 mL (Büchi Rotavapor R-134, Flawil, Switzerland), and diluted with water to 40 mL. This methanol-water 1:1 crude extract was purified by solid-phase extraction using Strata-XL cartridges (10 portions, 200 mg 100 µm polymeric RP, Phenomenex, Torrance, CA, USA). The cartridge was rinsed with 4 mL of methanol, conditioned with 4 mL of 50% aqueous methanol, loaded with 4 mL of sample, washed with 4 mL of acetonitrile, and then eluted with 4 mL of ethanol. The whole 10 eluates (from 10 cartridges) were pooled, concentrated by a rotary evaporator to 2 mL, and transferred to HPLC analysis.

### 3.6. Compound Isolation by HPLC–DAD–ESI-MS

The antioxidant compounds were isolated by HPLC using an LCMS-2020 system (Shimadzu, Kyoto, Japan) consisting of a binary gradient solvent pump, a vacuum degasser, a thermostated autosampler, a column oven, a photodiode detector, and a mass analyzer using an electrospray ionization (ESI) interface. The instrument control, data acquisition, and data processing were carried out by LabSolutions 5.42v software (Shimadzu). Separation was achieved on a Gemini C_18_ column (250 mm length, 4.6 mm ID, 5 µm particle size, Phenomenex, Torrance, CA, USA) at 35 °C with a linear gradient of 5% aqueous acetonitrile with 0.05% formic acid (A) and methanol with 0.05% formic acid (B). The gradient program was as follows: 0–16 min, 92% B; 16–25 min, 92–100% B; 25–35 min, 100% B; and 35.1–40 min, 92% B. The flow rate of the mobile phase was adjusted to 1.2 mL/min. The injection volume was set to 1 µL for method development and 100 µL for isolation. The appropriate peaks were collected based on the UV chromatogram at 323 nm, and the fractionation protocol was repeated 15 times. The combined 15 fractions were dried with a rotary evaporator at 40 °C and transferred to NMR spectroscopy. The MS conditions were as follows: nebulizer gas (N_2_) flow rate 1.5 L/min, drying gas (N_2_) flow rate 15 L/min, interface temperature 350 °C, heat block temperature 400 °C, desolvation line temperature 250 °C, and detector voltage 4.5 kV. Full mass scan spectra were recorded in the negative ionization mode in the range of *m*/*z* 150–1000 with a scan speed of 883 u/s.

### 3.7. NMR Spectroscopy

The isolated compounds **R1**–**R9** were dissolved in methanol-*d*_4_, and the samples were transferred to a standard 5 mm NMR tube for measurements. NMR spectra were collected on a Varian DDR 600 (^1^H: 599.9 MHz, ^13^C: 150.9 MHz; 14.1 T) spectrometer equipped with a dual 5 mm inverse-detection pulsed-field gradient (IDPFG) probehead at 298 K. The instrument was operated and controlled by VnmrJ 3.2C software. All applied pulse sequences were obtained from the Chempack 5.1 standard pulse program library of the instrument. ^1^H and ^13^C chemical shifts (*δ*) are provided on the *δ*-scale, reported in ppm and referenced to the NMR solvent used (CHD_2_OD residual peak at *δ*_H_ = 3.31 ppm and CD_3_OD at *δ*_C_ = 49.0 ppm), whereas spin-spin coupling constants (*J*) are given in Hz. The signal multiplicities are denoted as s—singlet, br s—broad singlet, d—doublet, t—triplet, p—pentet; m—multiplet; dd—doublet of doublets; td—triplet of doublets. The full ^1^H and ^13^C NMR resonance assignments were performed by means of comprehensive one- (^1^H and ^13^C) and two-dimensional homonuclear (^1^H–^1^H COSY and ^1^H–^1^H TOCSY) and heteronuclear (^1^H–^13^C edHSQC (^1^*J*_C–H_ = 140 Hz) and ^1^H–^13^C HMBC (*^n^J*_C–H_ = 8 Hz), both of them gradient-enhanced with adiabatic pulses) NMR experiments. In the case of compound **1**, band-selective HSQC (bsHSQC) and HMBC (bsHMBC) spectra were also recorded to enhance the spectral resolution in the F1 dimension.

### 3.8. ATR-FTIR Spectroscopy

The ATR-FTIR spectra were recorded by a Perkin Elmer Spectrum 400 FT-IR/FT-NIR spectrometer (Waltham, MA, USA) equipped with a diamond/ZnSe ATR crystal and a MIR TGS detector. Spectra were collected in the range of 4000–650 cm^−1^ with a spectral resolution of 4 cm^–1^. A few drops of the isolates (1 mg/mL in ethanol) were placed onto the ATR crystal, then the solvent was completely evaporated and the spectra were obtained by averaging 8–32 scans after background subtraction. Data processing and analysis were performed by Perkin Elmer Spectrum Software version 6.3.1, which included baseline correction and Savitzky-Golay smoothing.

### 3.9. GC–MS

The isolated compounds **R2**, **R4**, and **R6** were dissolved in ethanol (1 mg/mL). For the GC–MS analysis, a Shimadzu GCMS-TQ8040 NX instrument was applied using a Rtx-5 (30 m × 250 µm i.d.; film thickness: 0.32 µm, Restek, Bellefonte, PA, USA) capillary column. Helium was used as a carrier gas with a linear velocity of 50 cm/s. The solution of each compound (1 µL) was injected in split mode (split ratio 1:20) at 300 °C. The column oven temperature was programmed to increase from 80 °C to 320 °C at 20 °C/min, and the final temperature was held for 10.5 min. The ionization in the electron impact ion source was performed with an electron beam of 70 eV. The triple quadrupole analyzer operated in full scan mode (*m*/*z* range 29–600, scan speed 3333 amu/s). The interface and the ion source temperatures were maintained at 280 °C, and the accelerating and detector voltages were set to 4.0 kV and 0.9 kV, respectively. The data were acquired and evaluated with GCMSsolutions 4.52 software (Shimadzu). The identification of the compounds was aided by the NIST 17 mass spectral library.

### 3.10. DPPH• Microplate Assay of Isolated Compounds

The antioxidant activity of the isolated compounds (1 mg/mL in ethanol) was evaluated using 96-well microplates and expressed as caffeic acid equivalents (mg caffeic acid/mg isolates and mol caffeic acid equivalent/mol isolates). Caffeic acid (10, 9, 8, 7, 6, 5, 4, 3, 2, 1 µL, 1 mg/mL in ethanol) and isolated compounds (10 µL) were pipetted to the wells in triplicate (on two separate occasions). After evaporation of the ethanol, 100 µL of DPPH• solution (0.3 M in methanol) was added to each well. After incubating the microplate at 25 °C for 10 min in the dark, the deep-violet stable free radical DPPH• was reduced to the pale-yellow 2,2-diphenyl-1-picrylhydrazine in the presence of antioxidants, resulting in a decrease in absorbance measured at 517 nm (Clariostar^®^ Plus microplate reader, BMG LABTECH, Ortenberg, Germany).

## 4. Conclusions

This study identified nine antioxidant caffeate esters from the stem bark of *R. pseudoacacia* using RP-HPTLC–DPPH• assay, RP-HPTLC–UV/VIS/FLD–HESI-HRMS, HPLC–DAD–ESI-MS, GC–MS, ATR–FTIR, and NMR spectroscopy. It led to the identification of 3-*O*-caffeoyl oleanolic acid (**R1**), oleyl caffeate (**R2**), octadecyl caffeate (**R3**), gadoleyl caffeate (**R4**), eicosanyl caffeate (**R5**), (*Z*)-9-docosenyl caffeate (**R6**), docosyl caffeate (**R7**), tetracosyl caffeate (**R8**), and hexacosanyl caffeate (**R9**). This is the first report for natural compounds **R2**, **R4**, and **R6**, while **R3**, **R5**, **R7**, **R8**, and **R9** were obtained from this genus for the first time. The antioxidant effects of the isolated compounds were confirmed using the DPPH• microplate assay. The stem bark of black locust holds significant potential as a candidate for pharmaceutical applications, as the known isolates display a range of other bioactivities such as antimicrobial, cytotoxic, antiproliferative, and anti-inflammatory properties.

## Figures and Tables

**Table 1:** Antioxidant compounds (**R1**–**R9**) isolated from the bark extract of black locust detected by RP-HPTLC–DPPH•–Vis and characterized by RP-HPTLC–HRMS.

Isolates	*hR*_F_	Observed *m*/*z* [M−H]^−^	Theoretical *m*/*z* [M−H]^−^	Error (ppm)	Proposed Molecular Formula	Isolated Amount (mg)	Assignment
**R** **1**	68	617.3848	617.3848	0.1	C_39_H_54_O_6_	2.1	3-*O*-caffeoyl oleanolic acid
**R** **2**	54	429.3005	429.3010	−1.2	C_27_H_42_O_4_	1.3	oleyl caffeate
**R** **3**	46	431.3162	431.3167	−1.2	C_27_H_44_O_4_	2.3	octadecyl caffeate
**R** **4**	42	457.3318	457.3323	−1.1	C_29_H_46_O_4_	2.0	gadoleyl caffeate
**R** **5**	33	459.3475	459.3480	−1.1	C_29_H_48_O_4_	1.7	eicosanyl caffeate
**R** **6**	37	485.3631	485.3636	−1.0	C_31_H_50_O_4_	0.6	(*Z*)-9-docosenyl caffeate
**R** **7**	28	487.3788	487.3793	−1.0	C_31_H_52_O_4_	1.4	docosyl caffeate
**R** **8**	22	515.4101	515.4106	−1.0	C_33_H_56_O_4_	0.9	tetracosyl caffeate
**R** **9**	16	543.4414	543.4419	−0.9	C_35_H_60_O_4_	0.9	hexacosanyl caffeate

**Table 2:** ^1^H and ^13^C NMR (CD_3_OD, 600/151 MHz) resonance assignments of 3-*O*-caffeoyl oleanolic acid (**R1**).

Position	*δ*_H_ (*J* in Hz)	*δ*_C_, Type
1a	1.69 (m, 1H)	39.4, CH_2_
1b	1.10 (m, 1H)	
2a	1.72 (m, 1H)	24.7, CH_2_
2b	1.67 (m, 1H)	
3	4.57 (dd, *J* = 11.5, 4.3 Hz, 1H)	82.3, CH
4	-	39.0, C
5	0.91 (m, 1H)	56.8, CH
6a	1.59 (m, 1H)	19.4, CH_2_
6b	1.47 (m, 1H)	
7a	1.56 (m, 1H)	33.9, CH_2_
7b	1.34 (m, 1H)	
8	-	40.6, C
9	1.66 (m, 1H)	49.1, CH
10	-	38.2, C
11	1.92 (m, 2H)	24.5, CH_2_
12	5.26 (t, *J* = 3.6 Hz, 1H)	123.5, CH
13	-	145.3, C
14	-	42.9, C
15a	1.79 (m, 1H)	28.9, CH_2_
15b	1.09 (m, 1H)	
16a	2.02 (td, *J* = 13.5, 3.5 Hz, 1H)	24.1, CH_2_
16b	1.60 (m, 1H)	
17	-	47.7, C
18	2.86 (dd, *J* = 13.9, 4.6 Hz, 1H)	42.8, CH
19a	1.70 (m, 1H)	47.3, CH_2_
19b	1.14 (m, 1H)	
20	-	31.6, C
21a	1.40 (td, *J* = 13.8, 3.8 Hz, 1H)	34.9, CH_2_
21b	1.20 (m, 1H)	
22a	1.77 (m, 1H)	33.9, CH_2_
22b	1.55 (m, 1H)	
23	0.91 (s, 3H)	28.7, CH_3_
24	0.97 (s, 3H)	17.3, CH_3_
25	1.01 (s, 3H)	15.9, CH_3_
26	0.84 (s, 3H)	17.7, CH_3_
27	1.19 (s, 3H)	26.4, CH_3_
28	-	182.1, C
29	0.91 (s, 3H)	33.6, CH_3_
30	0.95 (s, 3H)	24.0, CH_3_
1′	7.03 (d, *J* = 2.0 Hz, 1H)	115.1, CH
2′	-	146.7, C
3′	-	149.6, C
4′	6.78 (d, *J* = 8.2 Hz, 1H)	116.5, CH
5′	6.94 (dd, *J* = 8.2, 2.1 Hz, 1H)	122.9, CH
6′	-	127.8, C
7′	7.52 (d, *J* = 15.8 Hz, 1H)	146.7, CH
8′	6.24 (d, *J* = 15.9 Hz, 1H)	115.6, CH
9′	-	169.2, C

**Table 3:** Antioxidant activity of the isolated fatty alcohol caffeates expressed as caffeic acid equivalents (mean of triplicates with standard deviation SD).

Isolate	Mass Equivalency Caffeic Acid/Isolate (mg/mg ± SD)	Molar Equivalency Caffeic Acid/Isolate (mol/mol ± SD)
**R** **1**	0.35 ± 0.008	1.20 ± 0.024
**R** **2**	0.20 ± 0.005	0.49 ± 0.011
**R** **3**	0.19 ± 0.003	0.47 ± 0.007
**R** **4**	0.24 ± 0.002	0.61 ± 0.005
**R** **5**	0.26 ± 0.007	0.67 ± 0.018
**R** **6**	0.13 ± 0.003	0.35 ± 0.007
**R** **7**	0.17 ± 0.007	0.47 ± 0.021
**R** **8**	0.10 ± 0.003	0.29 ± 0.008

## Data Availability

Data are available upon reasonable request.

## Supplementary Materials

The following supporting information can be downloaded at: https://www.mdpi.com/article/10.3390/molecules29235673/s1, Figure S1: UV-VIS spectra (190–600 nm) of the compounds **R1–R9** recorded by RP-HPTLC-densitometry; Figure S2: HPTLC chromatogram of black locust bark isolates (**R1–R9**) and extract (**E**) on RP18 plates with acetonitrile-ethanol 3:2 *V*/*V* after derivatization with natural product reagent at UV 365 nm; Figures S3–S35: 1D and 2D NMR spectra of compounds **R1–R9**; Figures S36–S41: ATR FTIR spectra of compounds **R1**–**R7**; Figure S42: The experimental EI-MS spectrum of compound **R2** and the theoretical EI-MS spectrum of oleyl alcohol; Figure S43: The experimental EI-MS spectrum of compound **R4** and the theoretical EI-MS spectrum of gadoleyl alcohol; Figure S44: The experimental EI-MS spectrum of compound **R6**; Figure S45: GC–MS TIC chromatograms of compounds **R2**, **R4**, and **R6**; Figure S46: Plot of the logarithmic retention time (*t*_R_) versus the number of carbon atoms for compounds **R2**, **R4**, and **R6**.
