# Supplementary material for: New Antioxidant Caffeate Esters of Fatty Alcohols Identified in Robinia pseudoacacia
Source: Molecules. 2024 Nov 30;29(23):5673. doi: 10.3390/molecules29235673 (PMC11643422; doi:10.3390/molecules29235673)
Supplement: Supplementary file 1 [file molecules-29-05673-s001.zip › molecules-3306748-supplementary.pdf]

Supplementary Material

New Antioxidant Caffeate Esters of Fatty Alcohols Identified in *Robinia pseudoacacia*

Ágnes M. Móricz [1,*], Márton Baglyas [1,2], András Darcsi [3], József Balla [4] and Gertrud E. Morlock [5]

[1] Plant Protection Institute, HUN-REN Centre for Agricultural Research, Fehérvári út 132–144,
1116 Budapest, Hungary; baglyas.marton@atk.hun-ren.hu
[2] Doctoral School, Semmelweis University, Üllői út 26, 1085 Budapest, Hungary
[3] Pharmaceutical Chemistry and Technology Department, National Center for Public Health and Pharmacy, Szabolcs utca 33, 1135 Budapest, Hungary; darcsi.andrew@gmail.com
[4] Department of Inorganic and Analytical Chemistry, Faculty of Chemical Technology and Biotechnology, Budapest University of Technology and Economics, Szent Gellért tér 4, 1111 Budapest, Hungary; balla.jozsef@vbk.bme.hu
[5] Institute of Nutritional Science, Chair of Food Science, Justus Liebig University Giessen, Heinrich-Buff-Ring 26–32, 35392 Giessen, Germany; gertrud.morlock@uni-giessen.de
* Correspondence: moricz.agnes@atk.hun-ren.hu

Table of contents

No.	Legend	Page
**Figure S1**	UV-VIS spectra (190-600 nm) of the compounds **R1-R9** recorded by HPTLC-densitometry.	S-4
**Figure S2**	HPTLC chromatogram of black locust bark isolates (**R1-R9**) and extract (**E**) after derivatization with natural product reagent at UV 365 nm. HPTLC separation was performed on RP18 plates with acetonitrile-ethanol 3:2 *V*/*V*.	S-4
**Figure S3**	^{1}H NMR spectrum of compound **1** (CD_3OD, 600 MHz).	S-5
**Figure S4**	^{13}C NMR spectrum of compound **1** (CD_3OD, 151 MHz).	S-5
**Figure S5**	^{1}H–^{1}H COSY NMR spectrum of compound **1** (CD_3OD, 600 MHz).	S-6
**Figure S6**	^{1}H–^{13}C edHSQC NMR spectrum of compound **1** (CD_3OD, 600 and 151 MHz).	S-6
**Figure S7**	^{1}H–^{13}C bsHSQC NMR spectrum of compound **1** (CD_3OD, 600 and 151 MHz).	S-7
**Figure S8**	^{1}H–^{13}C HMBC NMR spectrum of compound **1** (CD_3OD, 600 and 151 MHz).	S-7
**Figure S9**	^{1}H–^{13}C bsHMBC NMR spectrum of compound **1** (CD_3OD, 600 and 151 MHz).	S-8
**Figure S10**	^{1}H–^{1}H TOCSY NMR spectrum of compound **1** (CD_3OD, 600 MHz).	S-8
**Figure S11**	^{1}H NMR spectrum of compound **2** (CD_3OD, 600 MHz).	S-9

**Figure S12**	^1H–^1H COSY NMR spectrum of compound **2** (CD_3OD, 600 MHz).	S-9
**Figure S13**	^1H–^{13}C edHSQC NMR spectrum of compound **2** (CD_3OD, 600 and 151 MHz).	S-10
**Figure S14**	^1H–^{13}C HMBC NMR spectrum of compound **2** (CD_3OD, 600 and 151 MHz).	S-10
**Figure S15**	^1H NMR spectrum of compound **3** (CD_3OD, 600 MHz).	S-11
**Figure S16**	^1H–^1H COSY NMR spectrum of compound **3** (CD_3OD, 600 MHz).	S-11
**Figure S17**	^1H–^{13}C edHSQC NMR spectrum of compound **3** (CD_3OD, 600 and 151 MHz).	S-12
**Figure S18**	^1H–^{13}C HMBC NMR spectrum of compound **3** (CD_3OD, 600 and 151 MHz).	S-12
**Figure S19**	^1H NMR spectrum of compound **4** (CD_3OD, 600 MHz).	S-13
**Figure S20**	^1H–^1H COSY NMR spectrum of compound **4** (CD_3OD, 600 MHz).	S-13
**Figure S21**	^1H–^{13}C edHSQC NMR spectrum of compound **4** (CD_3OD, 600 and 151 MHz).	S-14
**Figure S22**	^1H–^{13}C HMBC NMR spectrum of compound **4** (CD_3OD, 600 and 151 MHz).	S-14
**Figure S23**	^1H NMR spectrum of compound **5** (CD_3OD, 600 MHz).	S-15
**Figure S24**	^1H–^1H COSY NMR spectrum of compound **5** (CD_3OD, 600 MHz).	S-15
**Figure S25**	^1H–^{13}C edHSQC NMR spectrum of compound **5** (CD_3OD, 600 and 151 MHz).	S-16
**Figure S26**	^1H–^{13}C HMBC NMR spectrum of compound **5** (CD_3OD, 600 and 151 MHz).	S-16
**Figure S27**	^1H NMR spectrum of compound **6** (CD_3OD, 600 MHz).	S-17
**Figure S28**	^1H–^1H COSY NMR spectrum of compound **6** (CD_3OD, 600 MHz).	S-17
**Figure S29**	^1H NMR spectrum of compound **7** (CD_3OD, 600 MHz).	S-18
**Figure S30**	^1H–^1H COSY NMR spectrum of compound **7** (CD_3OD, 600 MHz).	S-18
**Figure S31**	^1H–^{13}C edHSQC NMR spectrum of compound **7** (CD_3OD, 600 and 151 MHz).	S-19
**Figure S32**	^1H–^{13}C HMBC NMR spectrum of compound **7** (CD_3OD, 600 and 151 MHz).	S-19
**Figure S33**	^1H NMR spectrum of compound **8** (CD_3OD, 600 MHz).	S-20
**Figure S34**	^1H–^1H COSY NMR spectrum of compound **8** (CD_3OD, 600 MHz).	S-20
**Figure S35**	Partial ^1H NMR spectrum of compound **9** (CD_3OD, 600 MHz).	S-21
**Figure S36**	ATR FTIR spectrum of compound **R1**.	S-21
**Figure S37**	ATR FTIR spectrum of compound **R2**.	S-22
**Figure S38**	ATR FTIR spectrum of compound **R3**.	S-22
**Figure S39**	ATR FTIR spectrum of compound **R4**.	S-23
**Figure S40**	ATR FTIR spectrum of compound **R5**.	S-23
**Figure S41**	ATR FTIR spectrum of compound **R7**.	S-24
**Figure S42**	The experimental EI-MS spectrum of compound **R2** (a) and the theoretical EI-MS spectrum of oleyl alcohol (b).	S-24
**Figure S43**	The experimental EI-MS spectrum of compound **R4** (a) and the theoretical EI-MS spectrum of gadoleyl alcohol (b).	S-25
**Figure S44**	The experimental EI-MS spectrum of compound **R6**.	S-25
**Figure S45**	GC–MS TIC chromatograms of compounds **R2** (black), **R4** (magenta), and **R6** (blue). The retention times of **R2**, **R4**, and **R6** are 7.520, 8.434 and 9.309 min, respectively.	S-25

**Figure S46**	Plot of the logarithmic retention time (t_R) versus the number of carbon atoms for compounds **R2**, **R4**, and **R6** with the fitted line (slope: 0.0232, intercept: 0.4605, R^2 = 0.998).	S-26

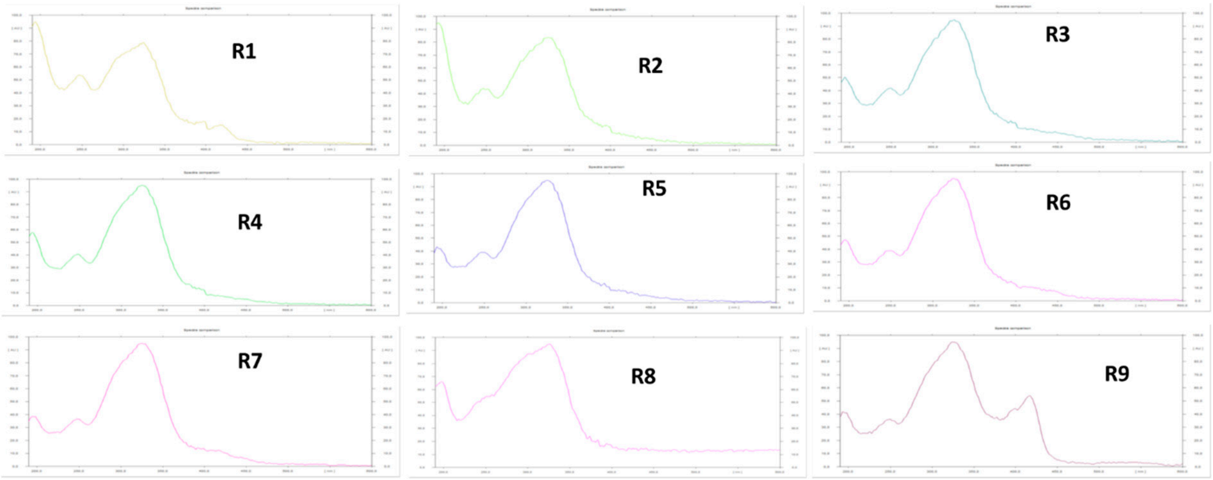

Figure S1. UV-VIS spectra (190-600 nm) of the compounds **R1**-**R9** recorded by HPTLC-densitometry.

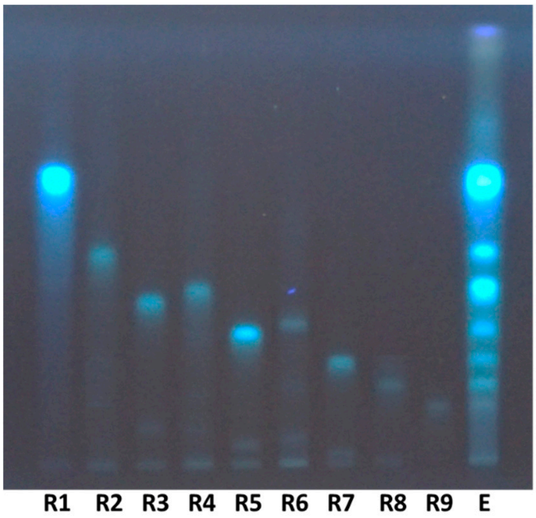

Figure S2. HPTLC chromatogram of black locust bark isolates (**R1**-**R9**) and extract (**E**) after derivatization with natural product reagent at UV 365 nm. HPTLC separation was performed on RP18 plates with acetonitrile-ethanol 3:2 *V*/*V*.

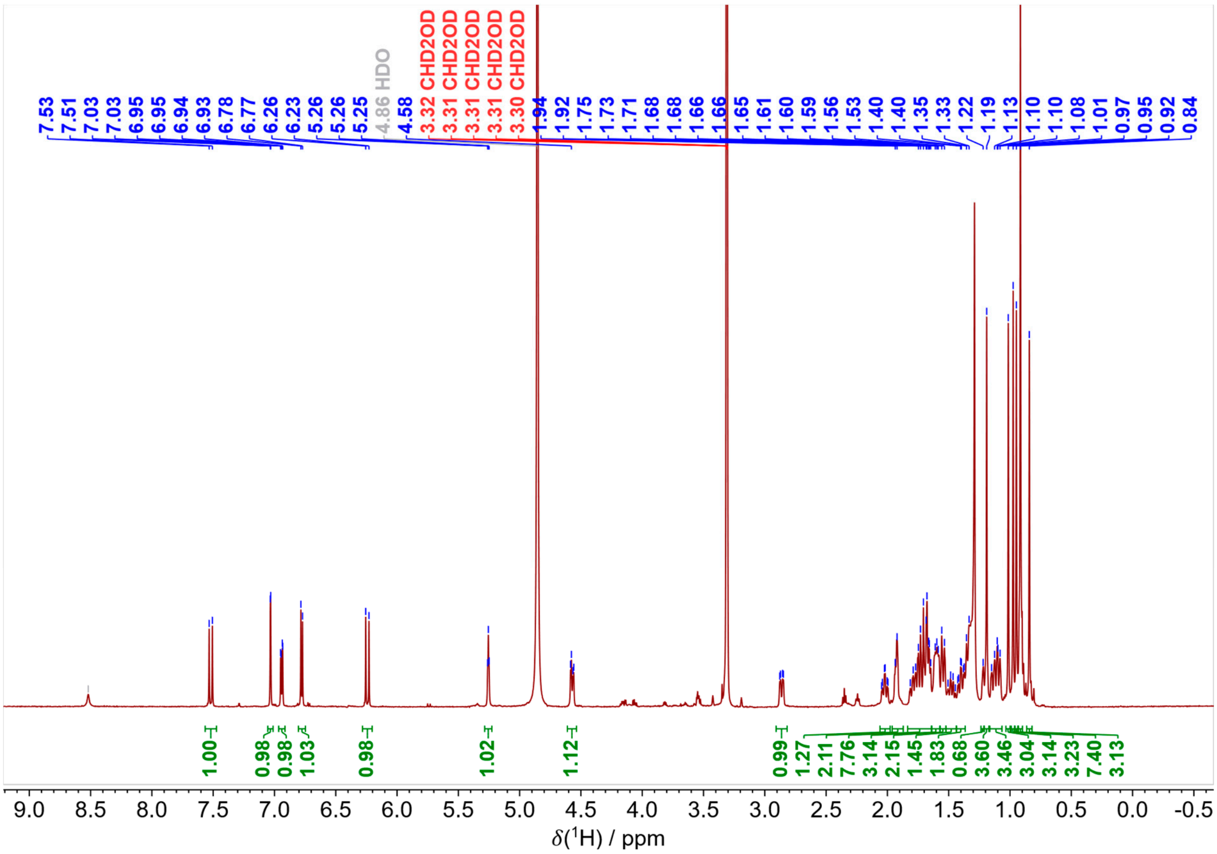

Figure S3. ^{1}H NMR spectrum of compound **1** (CD_3OD, 600 MHz).

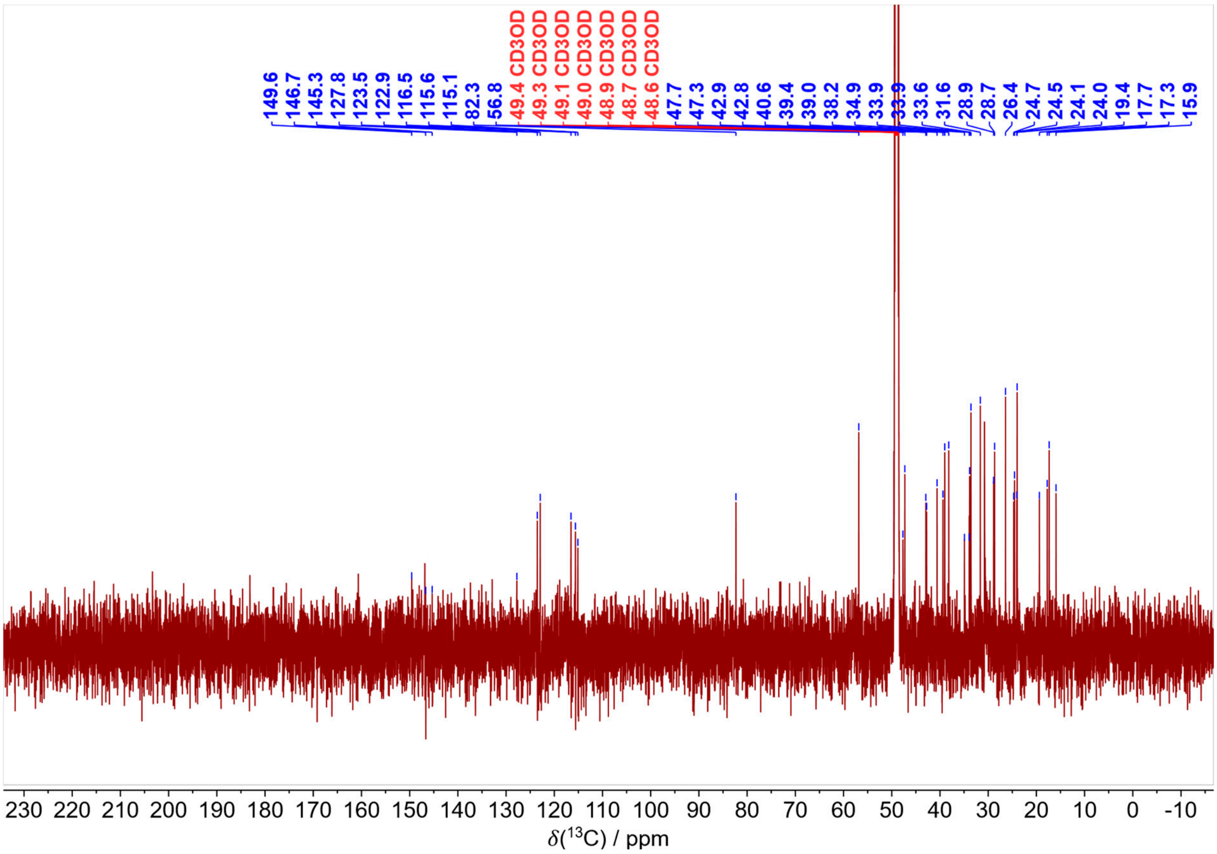

Figure S4. ^{13}C NMR spectrum of compound **1** (CD_3OD, 151 MHz).

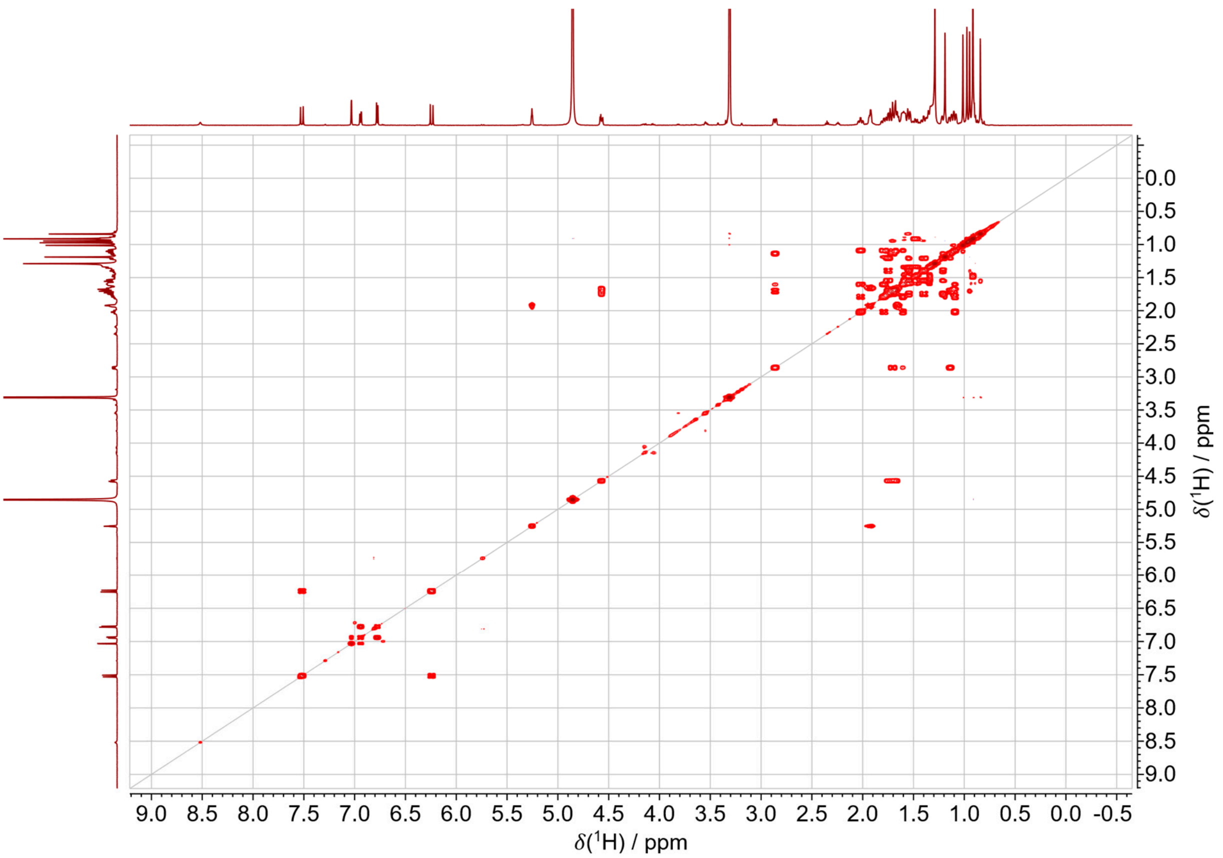

Figure S5. ^{1}H–^{1}H COSY NMR spectrum of compound **1** (CD_3OD, 600 MHz).

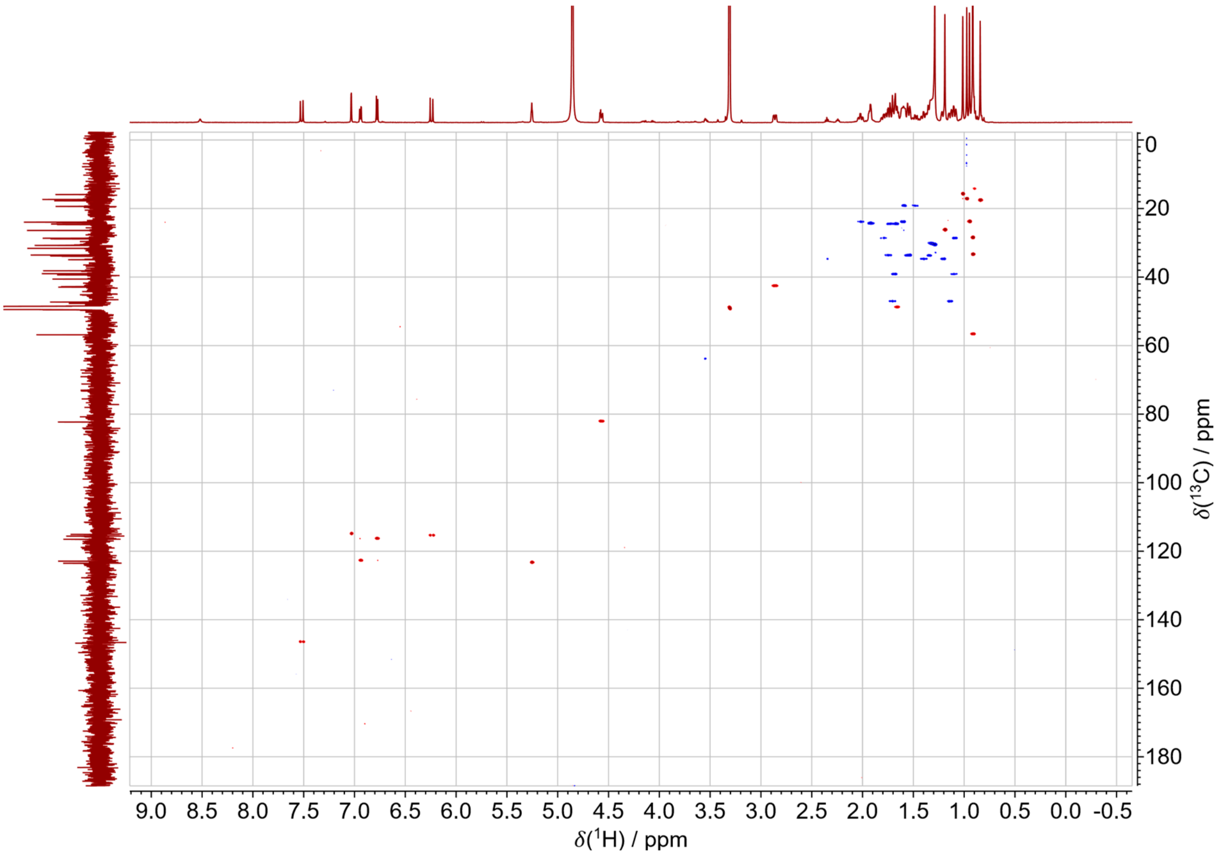

Figure S6. ^{1}H–^{13}C edHSQC NMR spectrum of compound **1** (CD_3OD, 600 and 151 MHz).

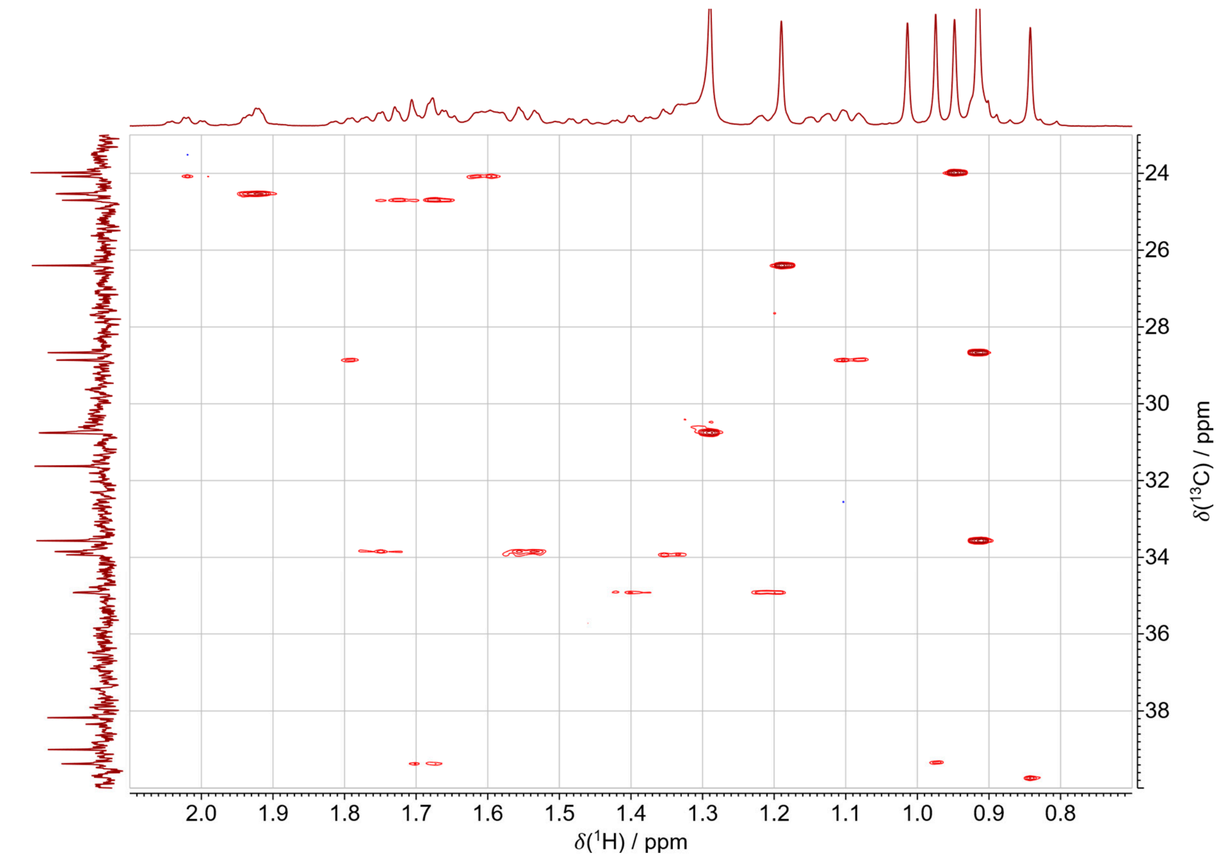

Figure S7. ^{1}H–^{13}C bsHSQC NMR spectrum of compound **1** (CD_3OD, 600 and 151 MHz).

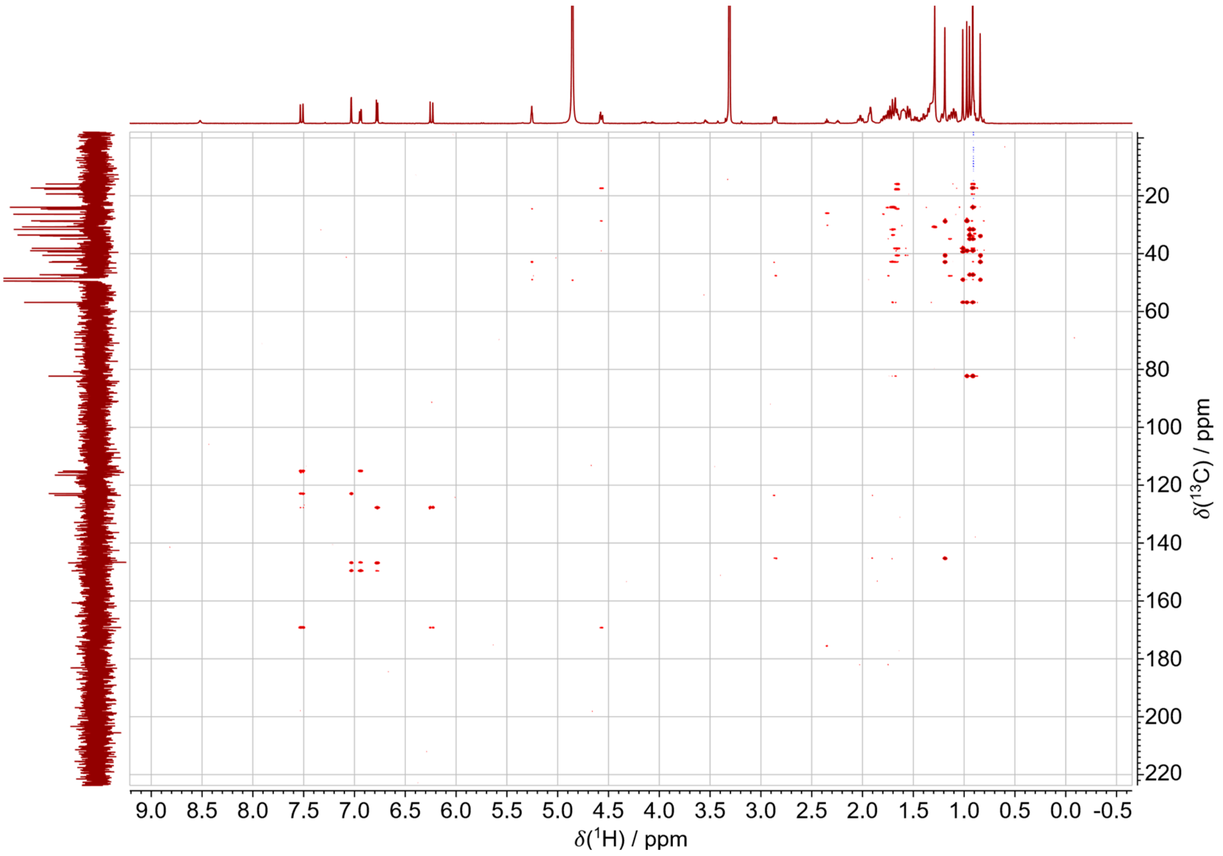

Figure S8. ^{1}H–^{13}C HMBC NMR spectrum of compound **1** (CD_3OD, 600 and 151 MHz).

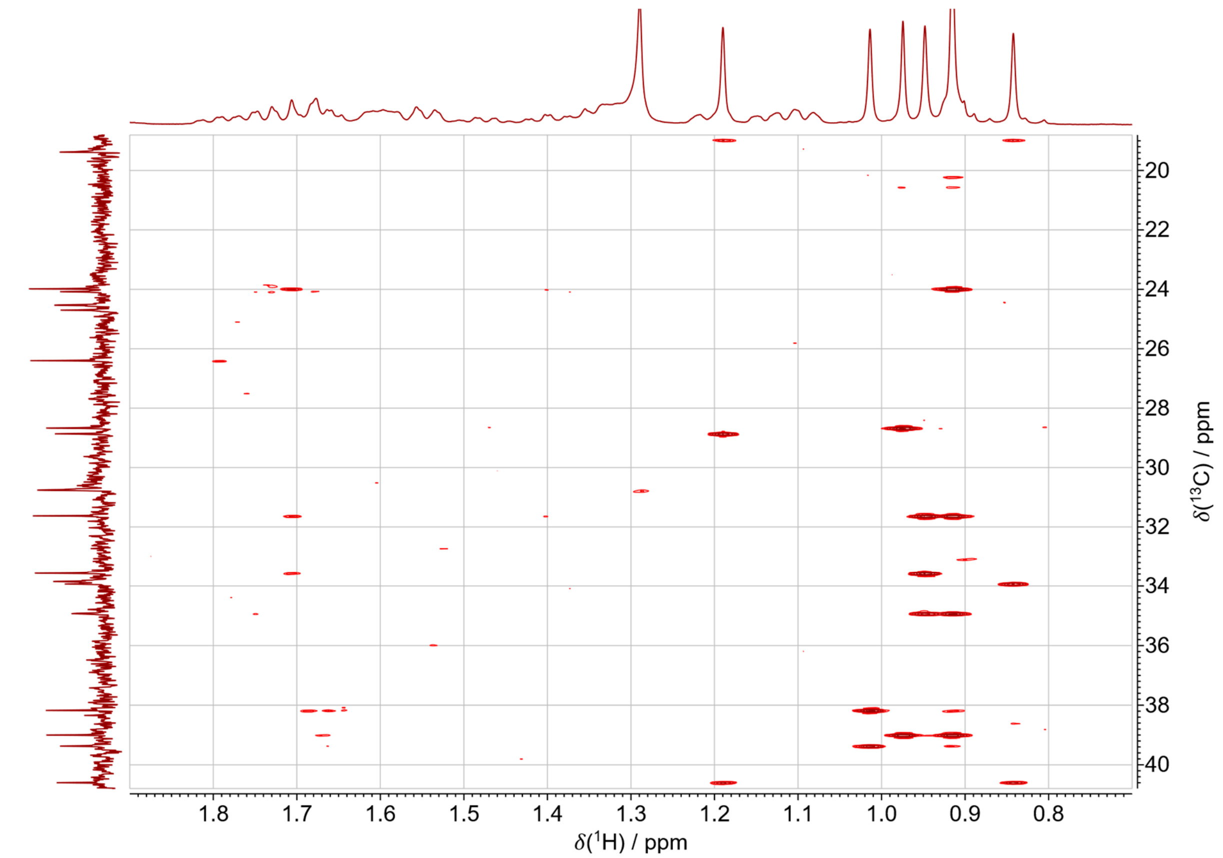

Figure S9. ^{1}H–^{13}C bsHMBC NMR spectrum of compound **1** (CD_3OD, 600 and 151 MHz).

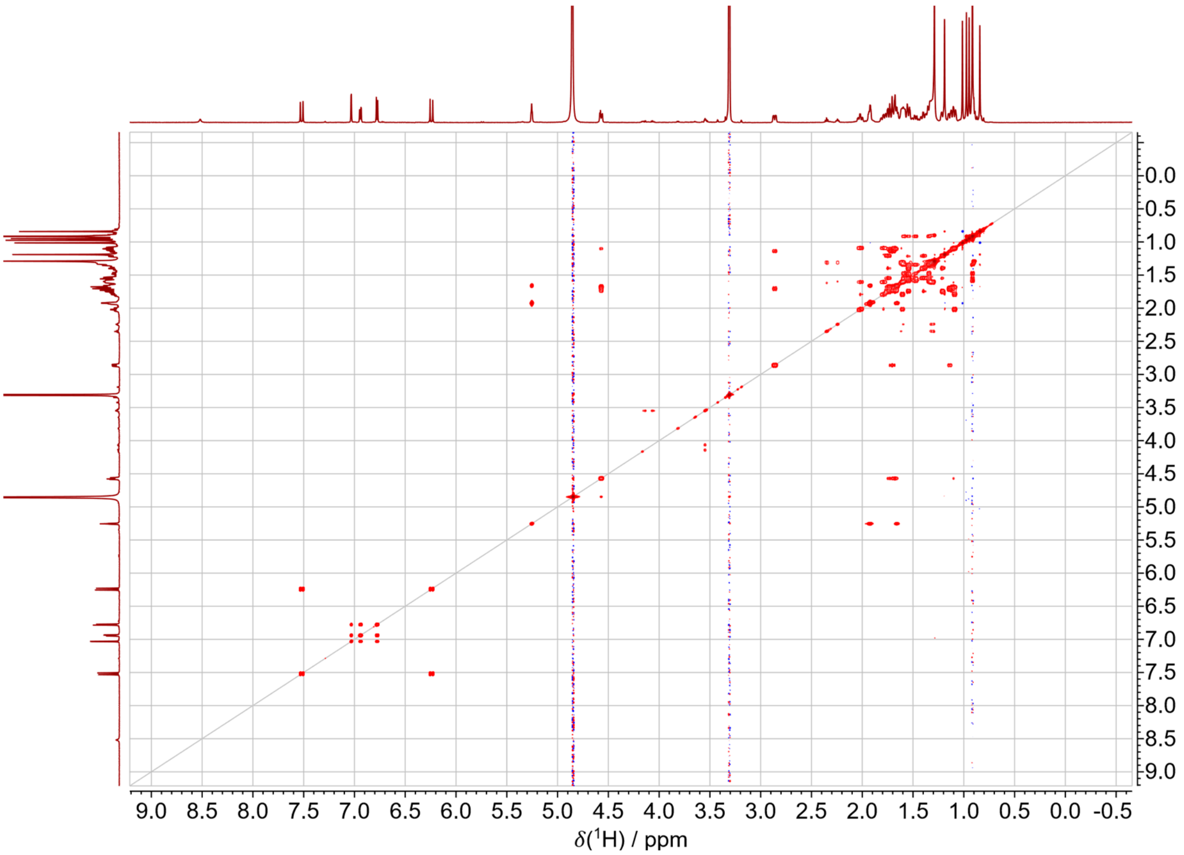

Figure S10. ^{1}H–^{1}H TOCSY NMR spectrum of compound **1** (CD_3OD, 600 MHz).

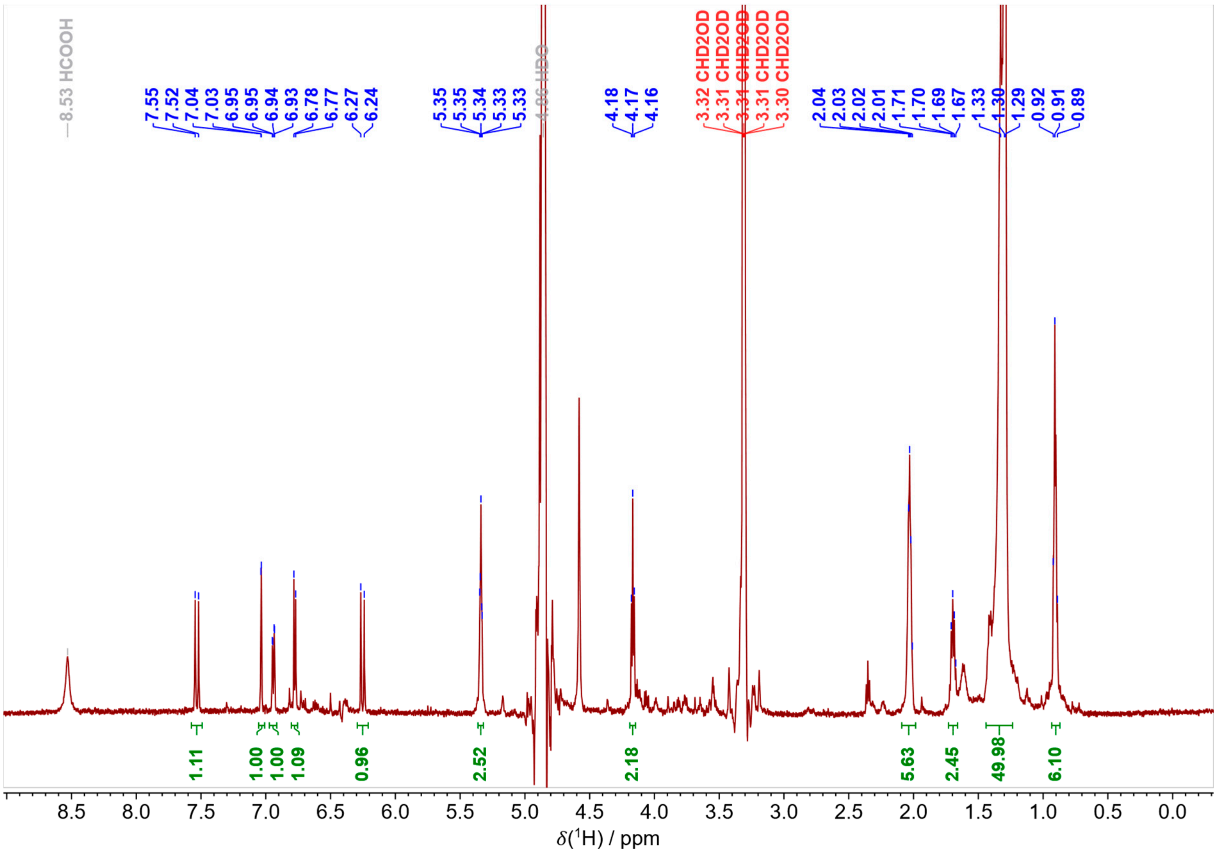

Figure S11. ^{1}H NMR spectrum of compound **2** (CD_3OD, 600 MHz).

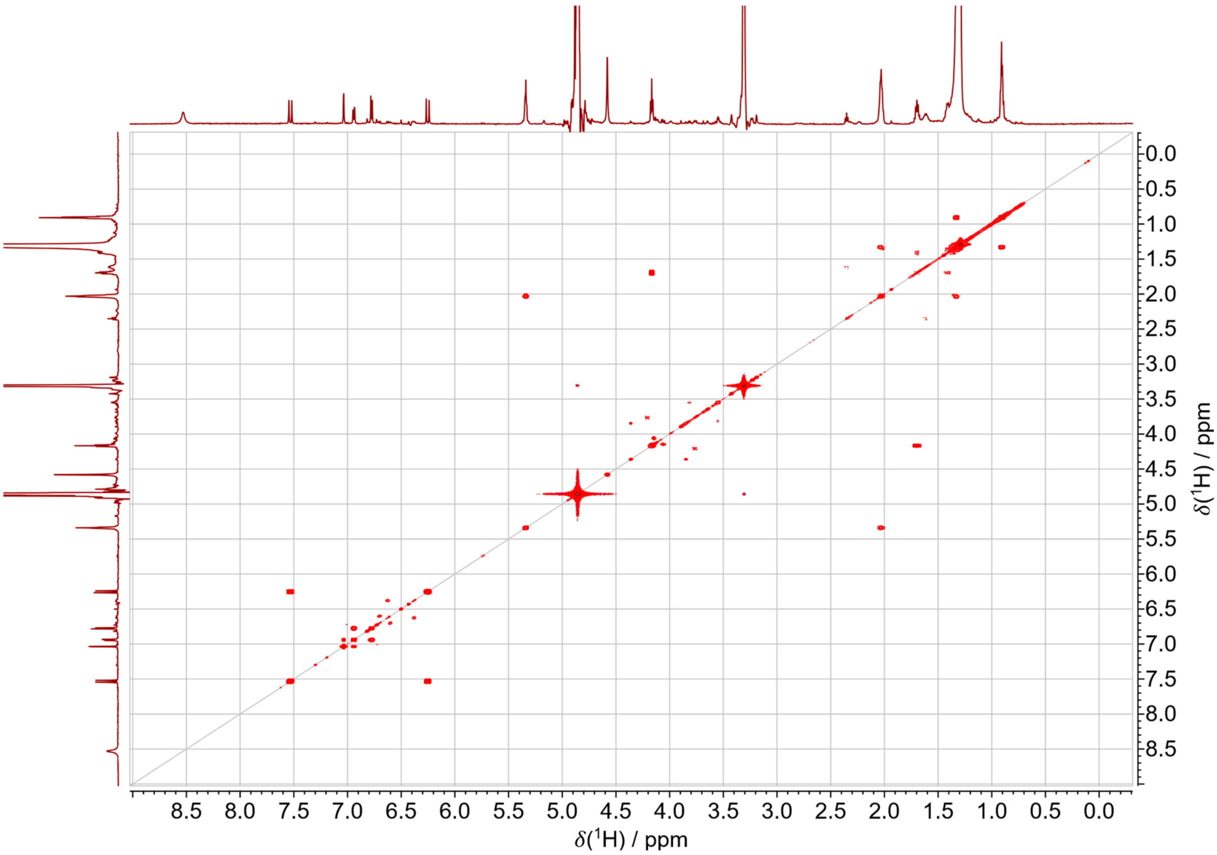

Figure S12. ^{1}H–^{1}H COSY NMR spectrum of compound **2** (CD_3OD, 600 MHz).

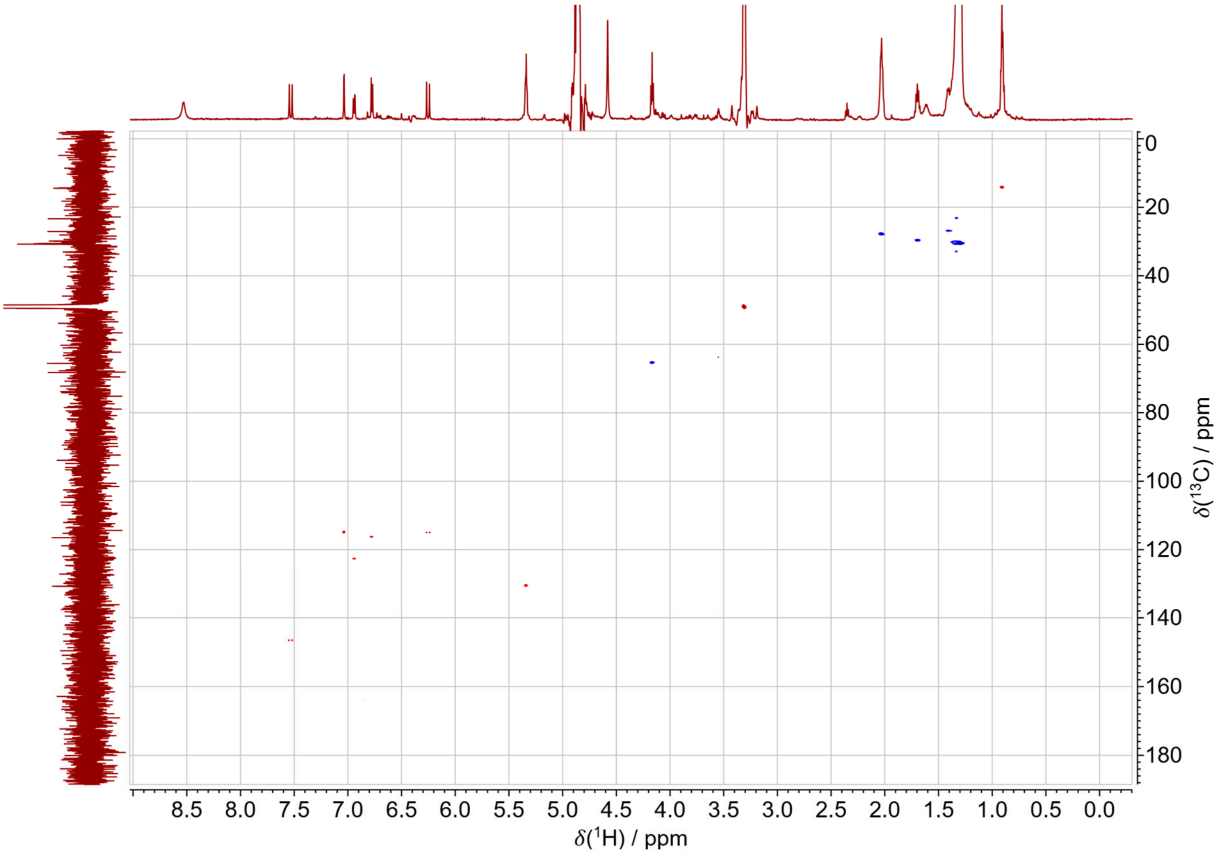

Figure S13. ^{1}H–^{13}C edHSQC NMR spectrum of compound **2** (CD_3OD, 600 and 151 MHz).

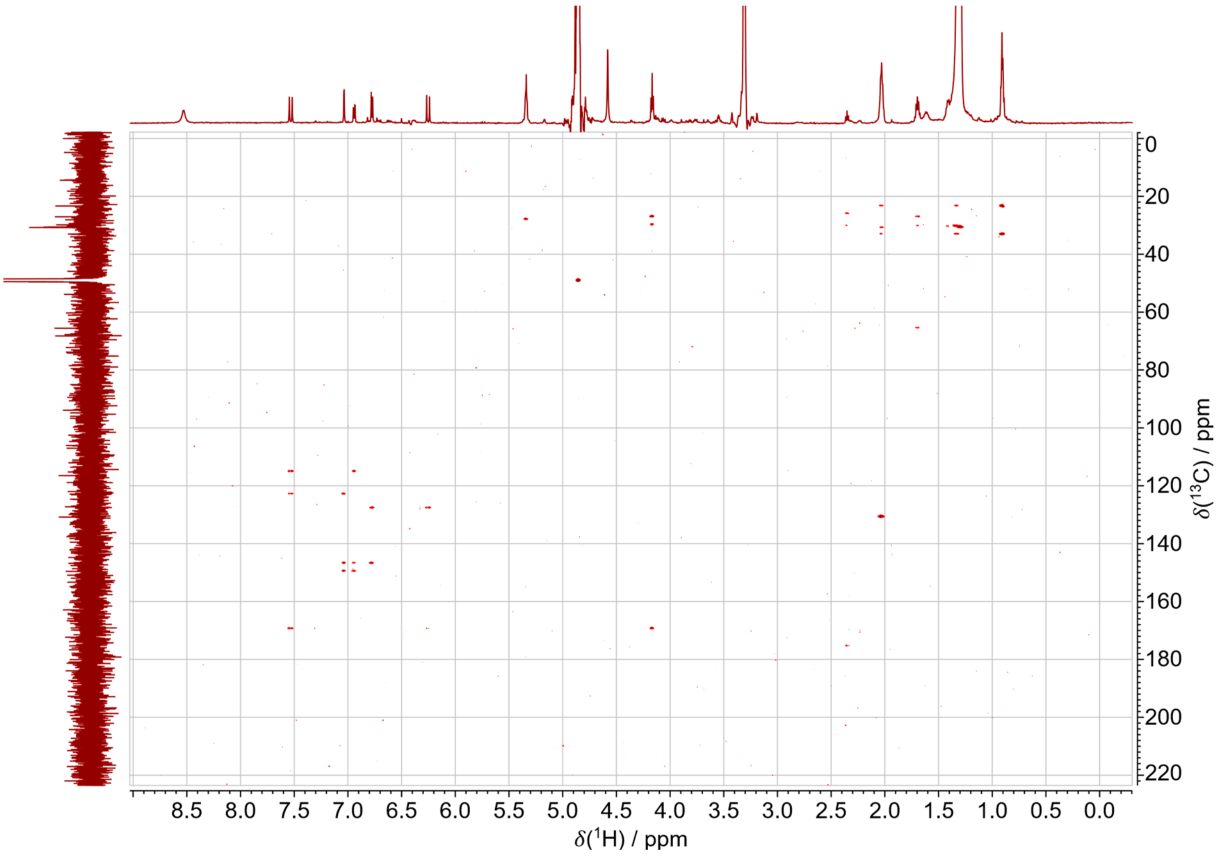

Figure S14. ^{1}H–^{13}C HMBC NMR spectrum of compound **2** (CD_3OD, 600 and 151 MHz).

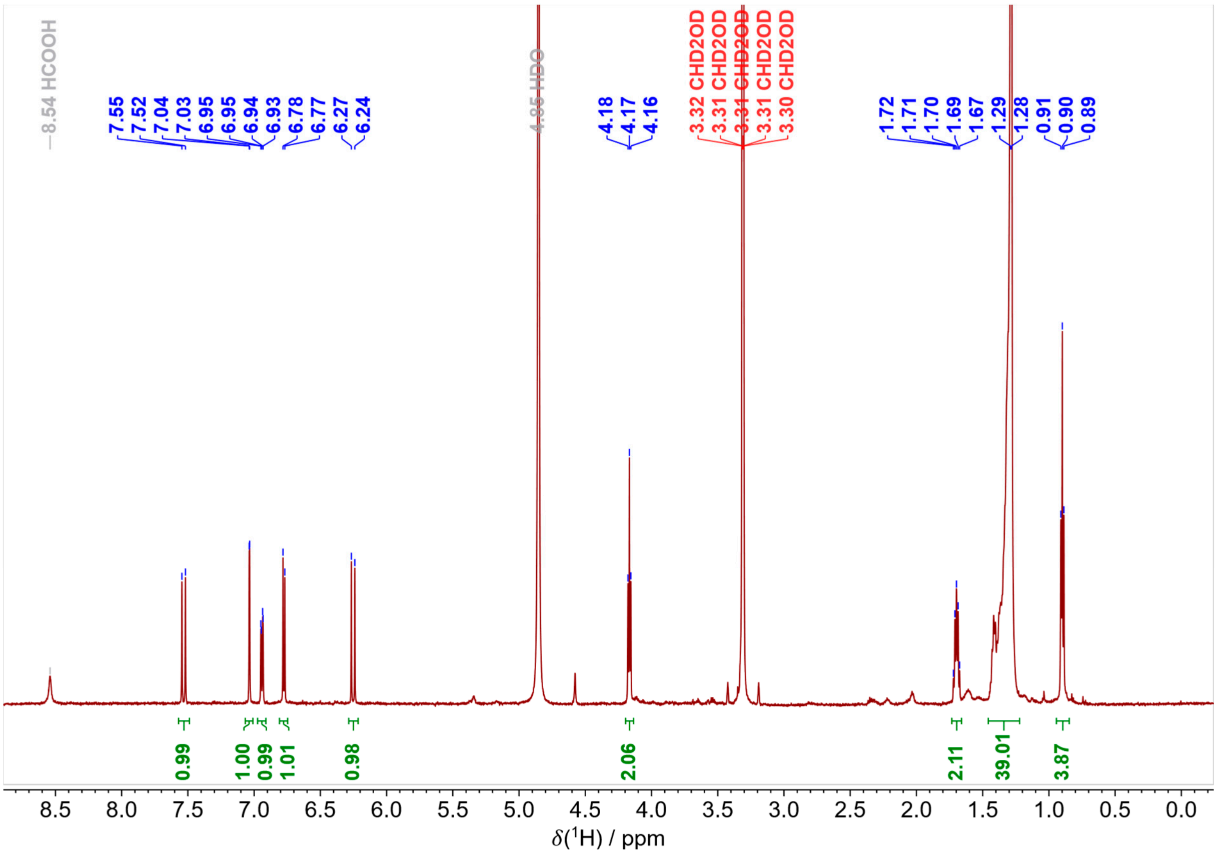

Figure S15. ^{1}H NMR spectrum of compound **3** (CD_3OD, 600 MHz).

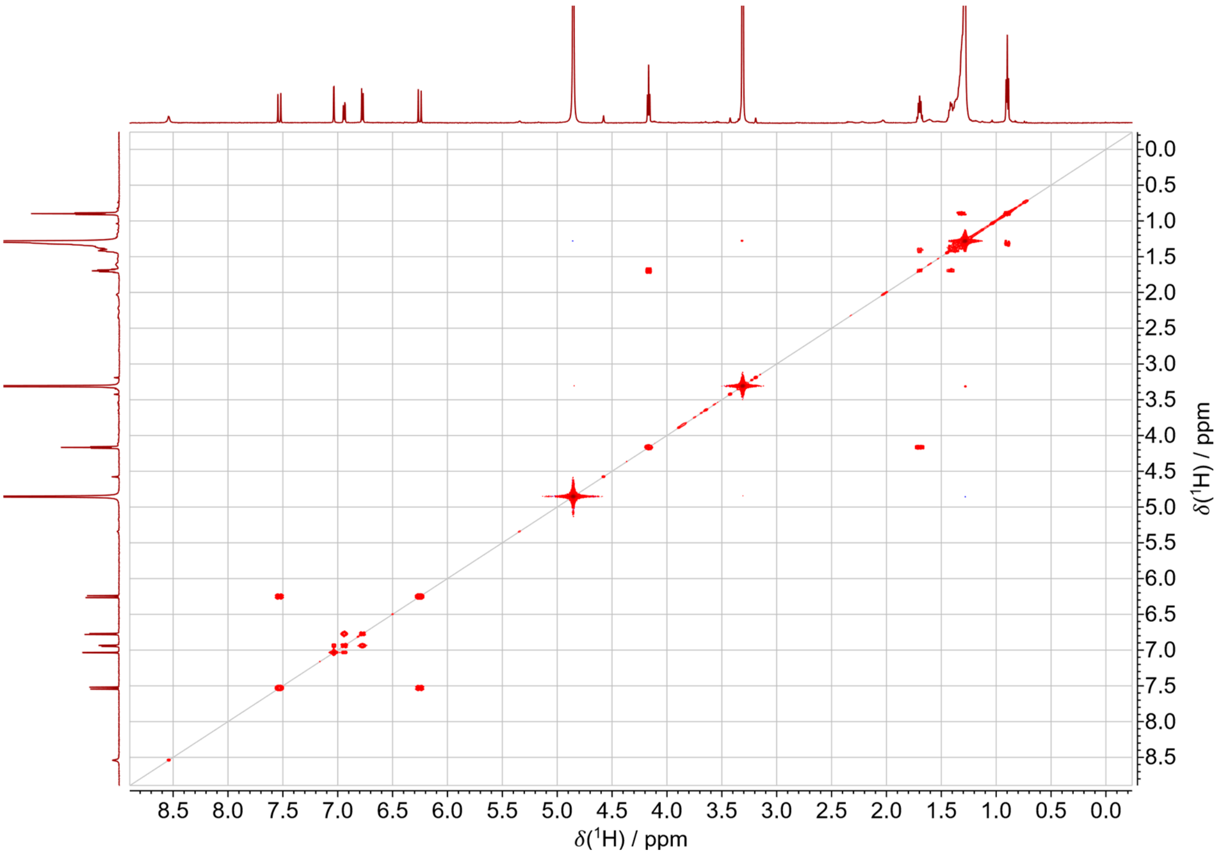

Figure S16. ^{1}H–^{1}H COSY NMR spectrum of compound **3** (CD_3OD, 600 MHz).

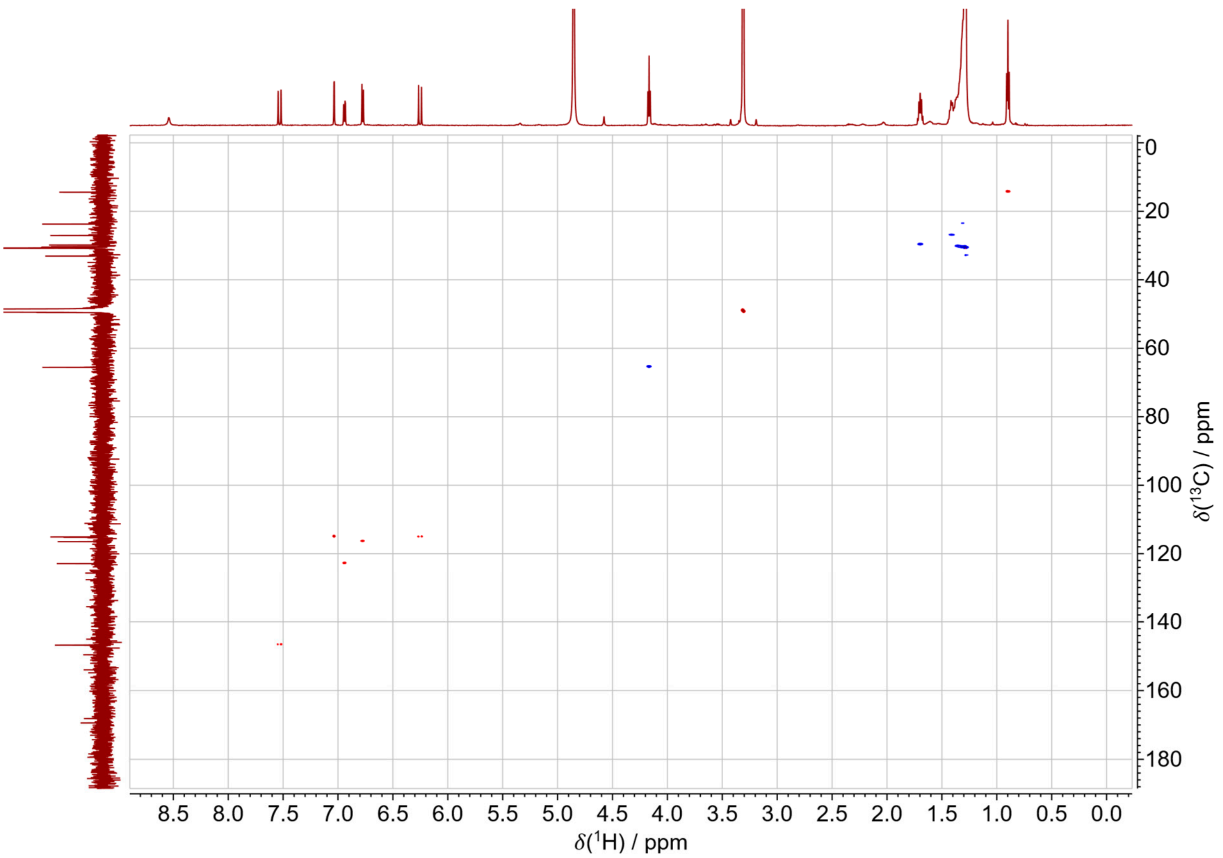

Figure S17. ^{1}H–^{13}C edHSQC NMR spectrum of compound **3** (CD_3OD, 600 and 151 MHz).

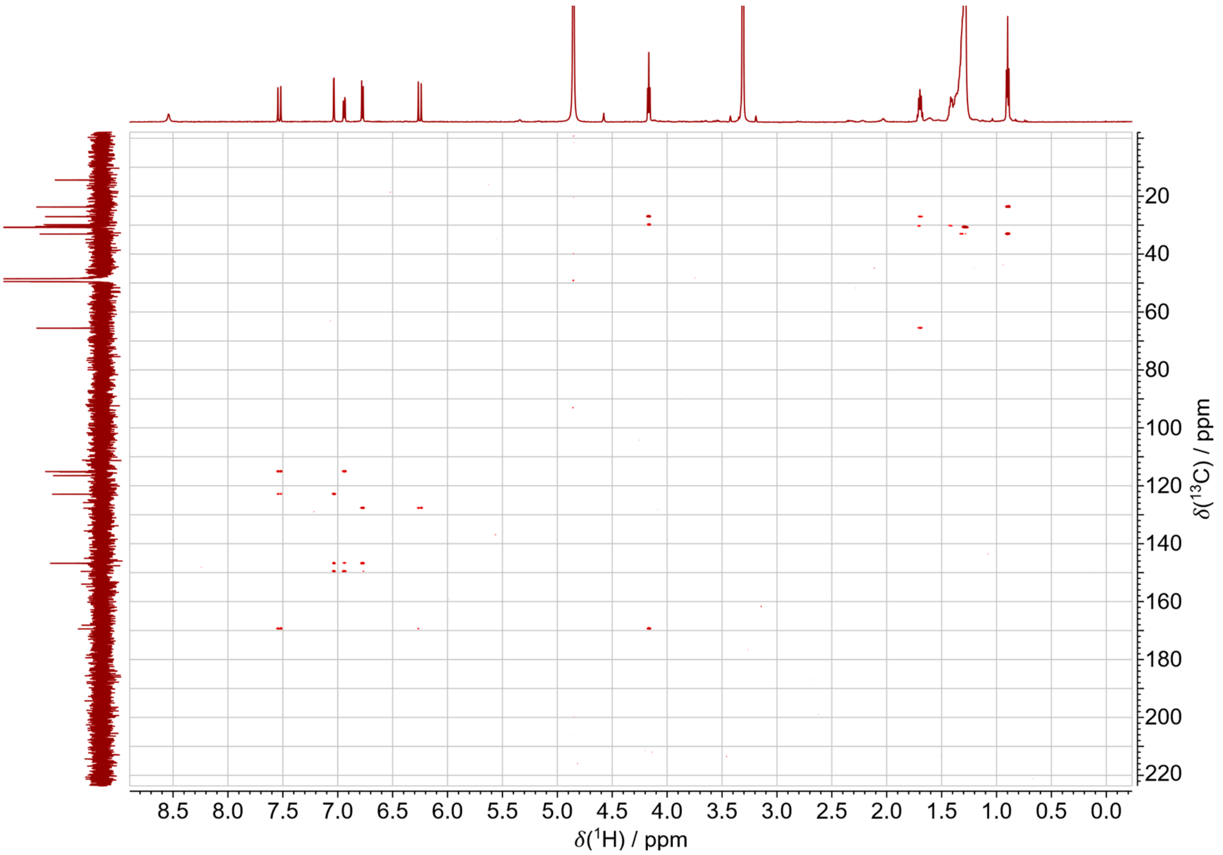

Figure S18. ^{1}H–^{13}C HMBC NMR spectrum of compound **3** (CD_3OD, 600 and 151 MHz).

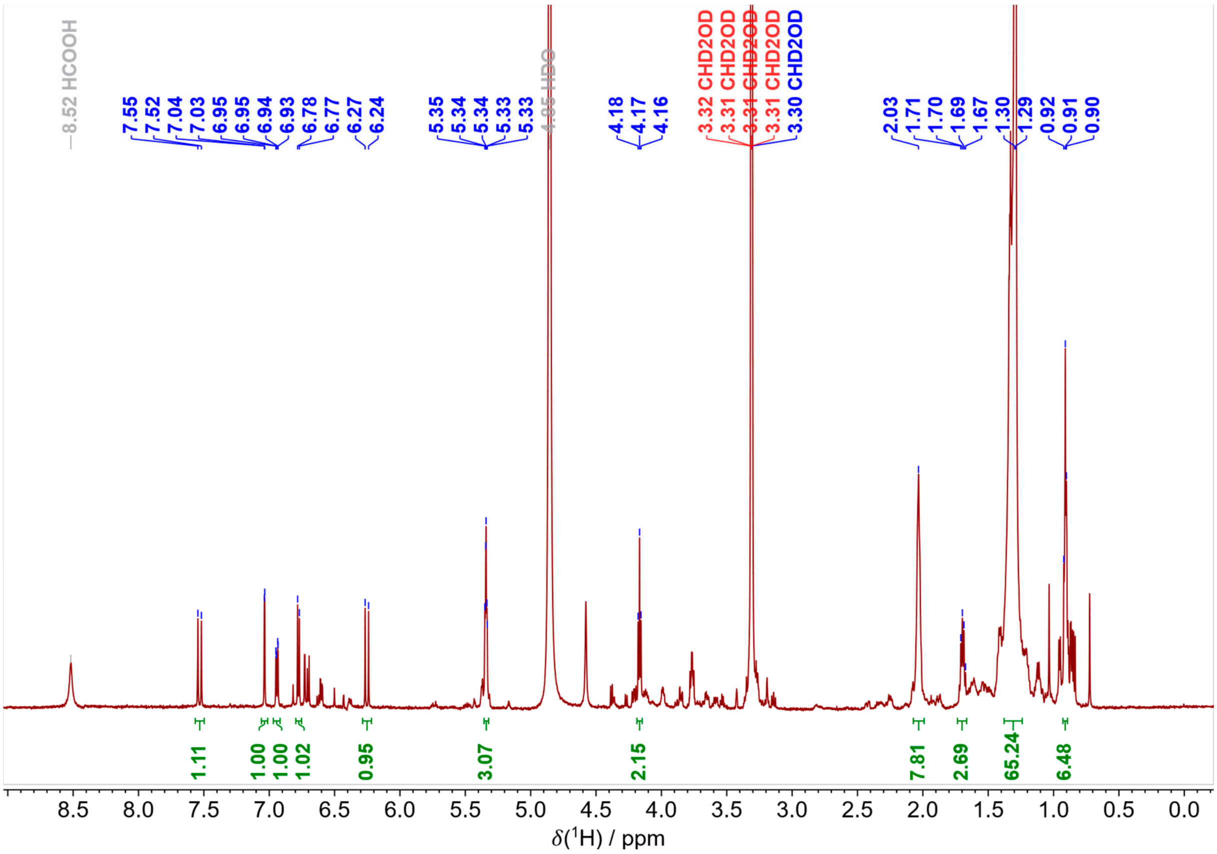

Figure S19. ^{1}H NMR spectrum of compound **4** (CD_3OD, 600 MHz).

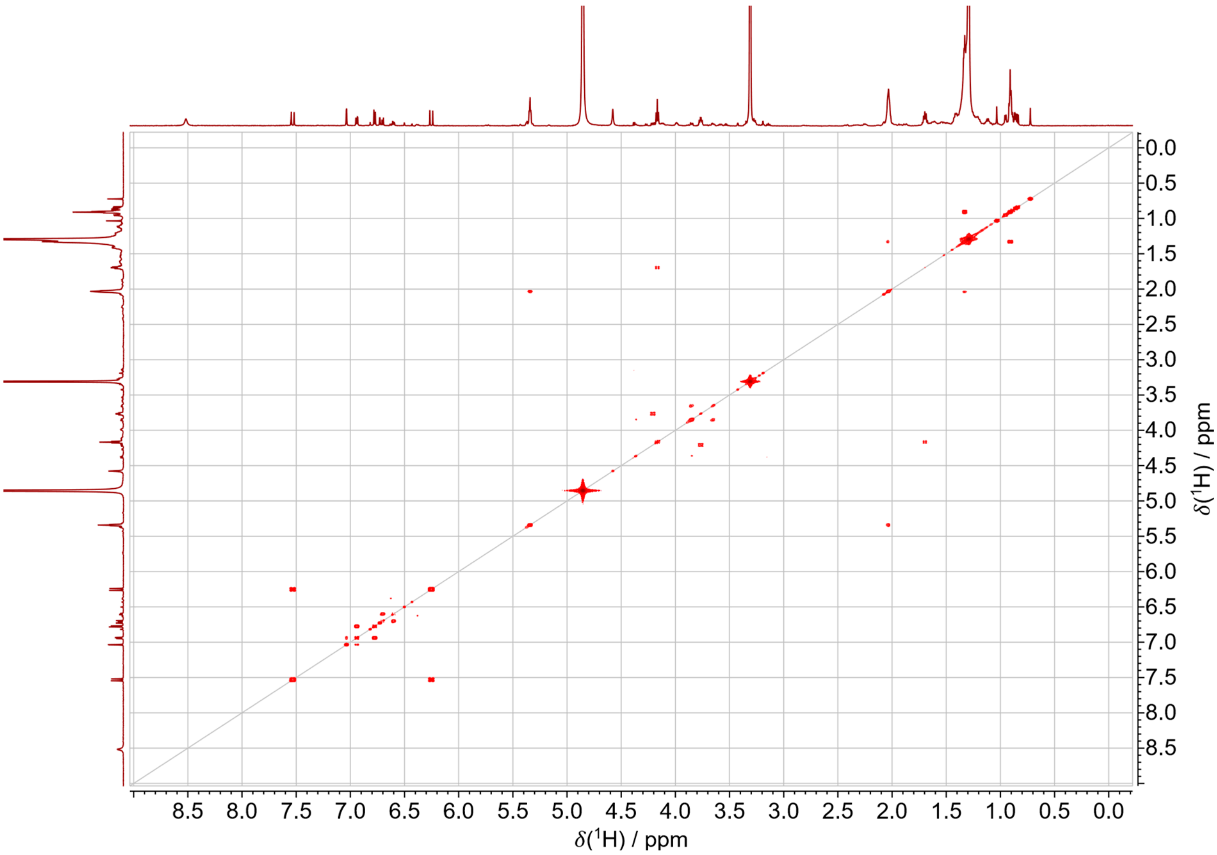

Figure S20. ^{1}H–^{1}H COSY NMR spectrum of compound **4** (CD_3OD, 600 MHz).

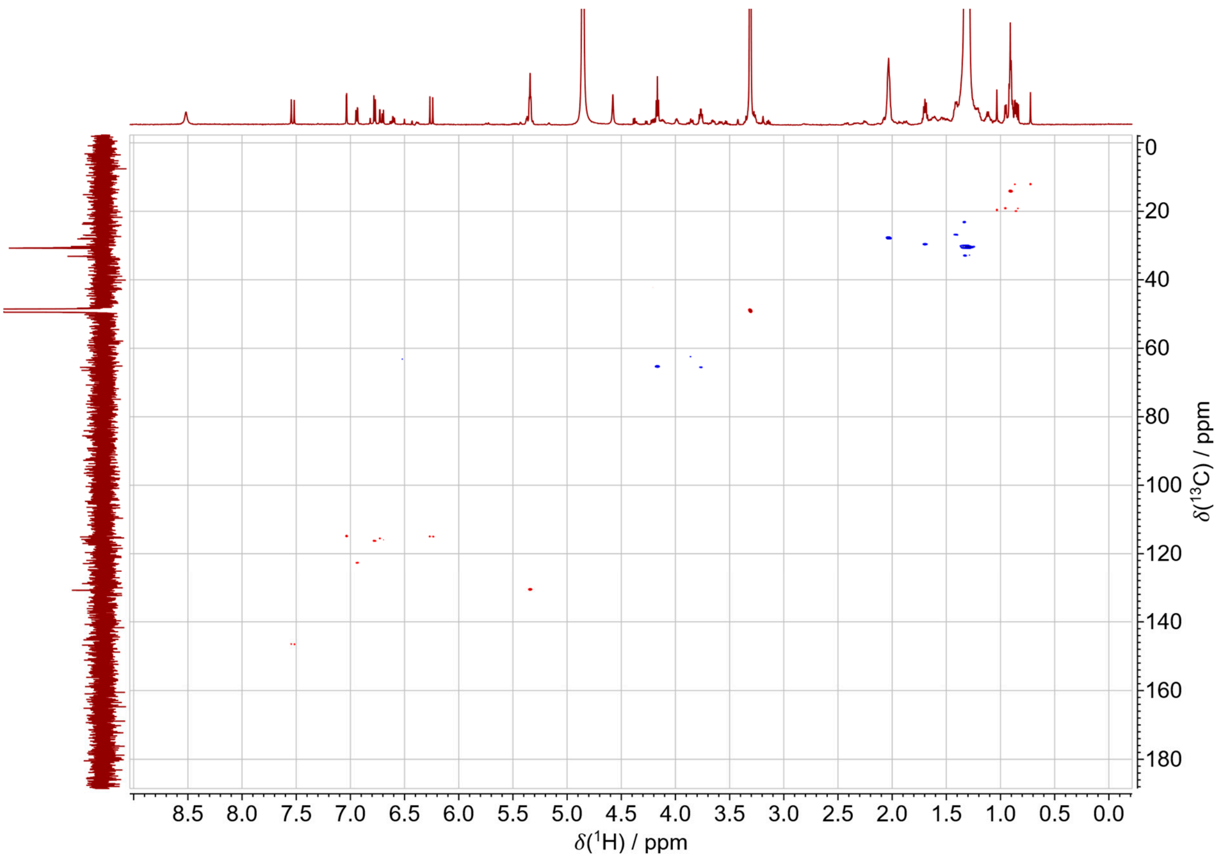

Figure S21. ^{1}H–^{13}C edHSQC NMR spectrum of compound **4** (CD_3OD, 600 and 151 MHz).

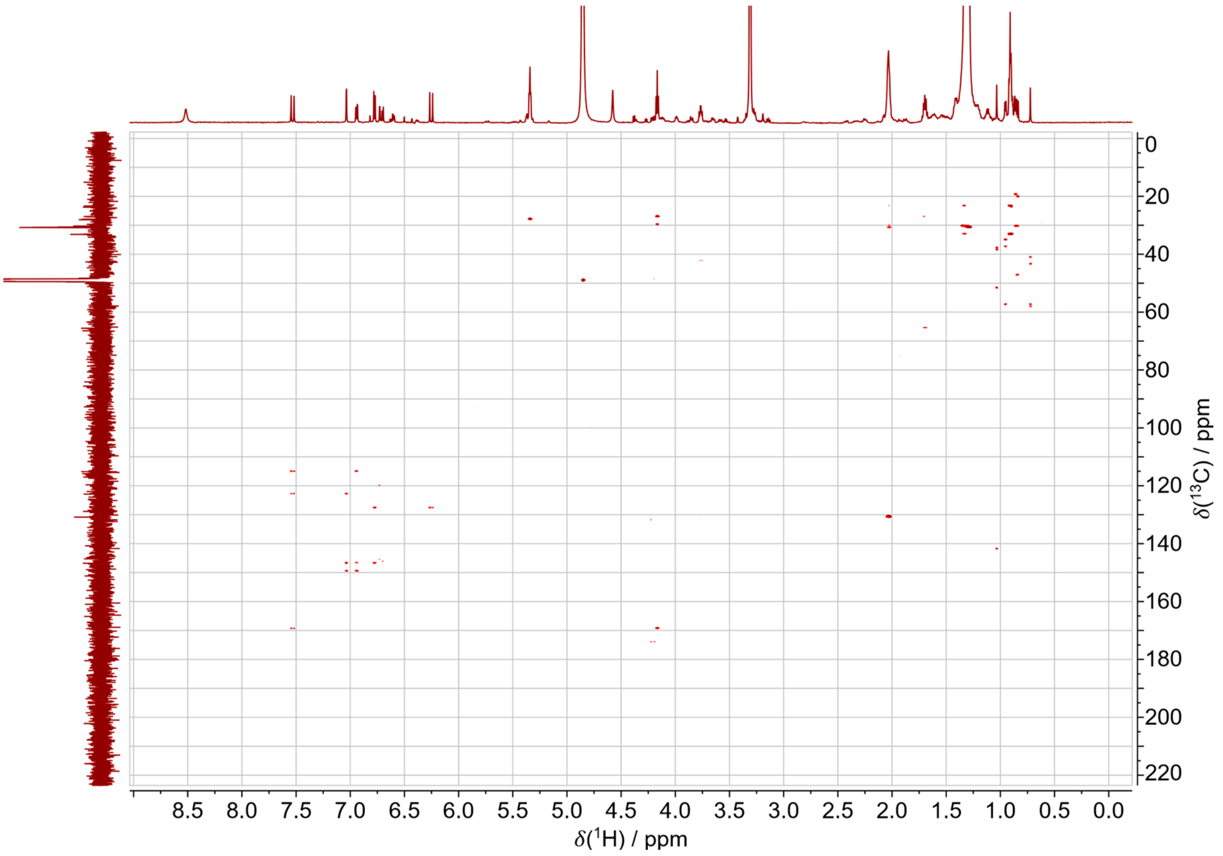

Figure S22. ^{1}H–^{13}C HMBC NMR spectrum of compound **4** (CD_3OD, 600 and 151 MHz).

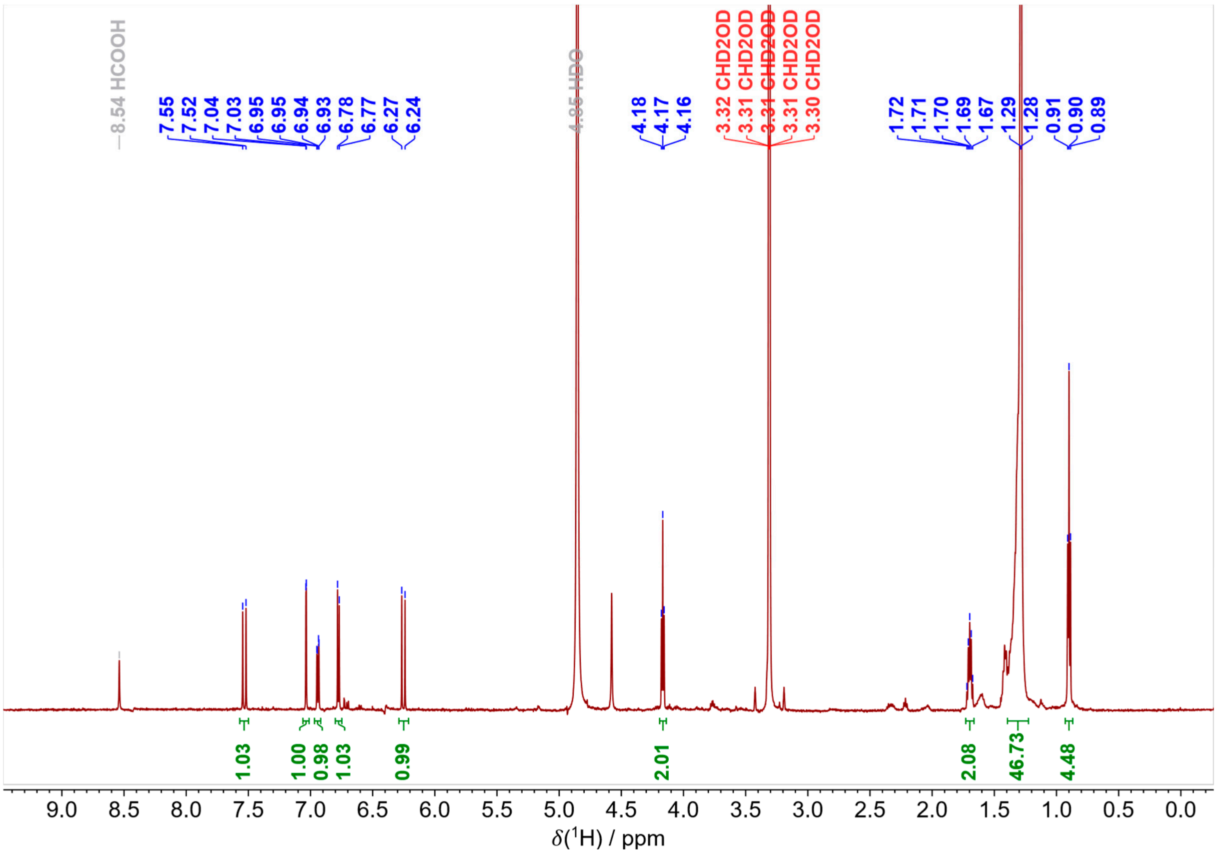

Figure S23. ^{1}H NMR spectrum of compound **5** (CD_3OD, 600 MHz).

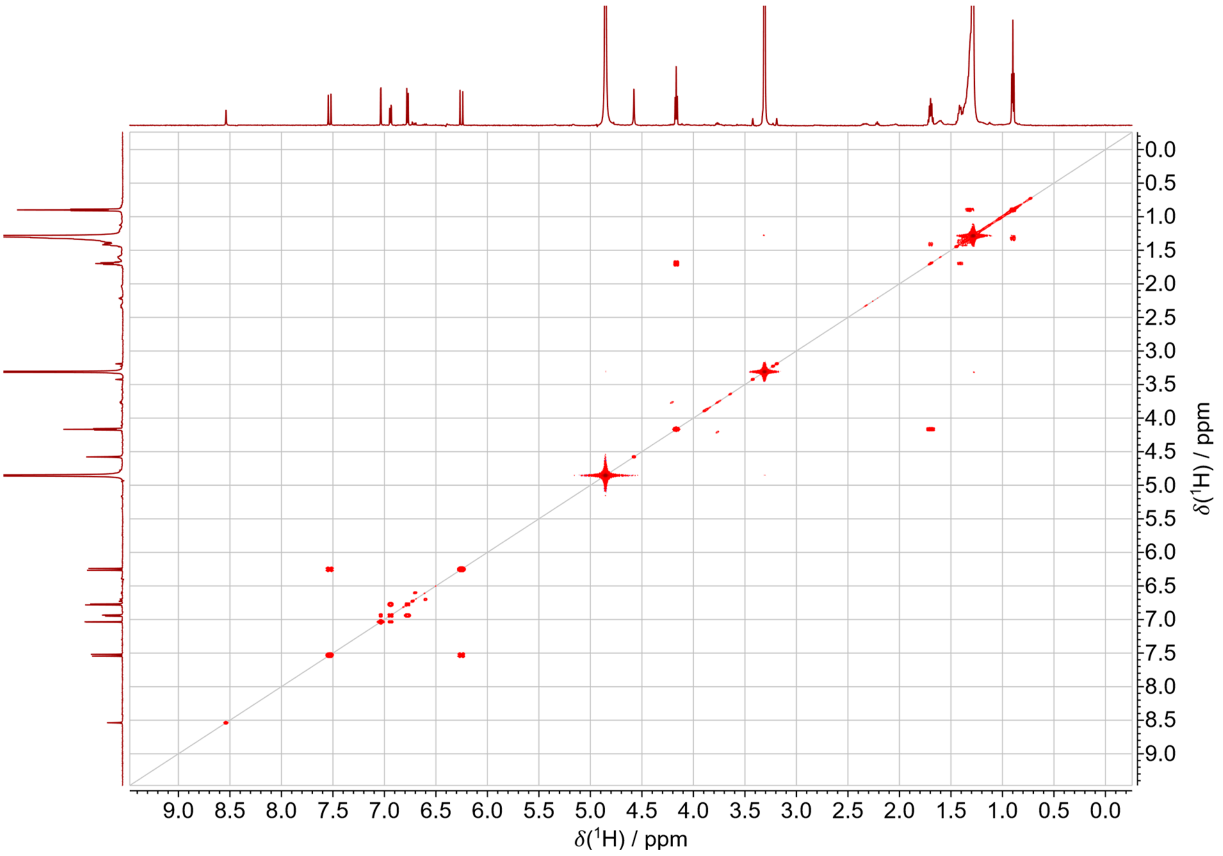

Figure S24. ^{1}H–^{1}H COSY NMR spectrum of compound **5** (CD_3OD, 600 MHz).

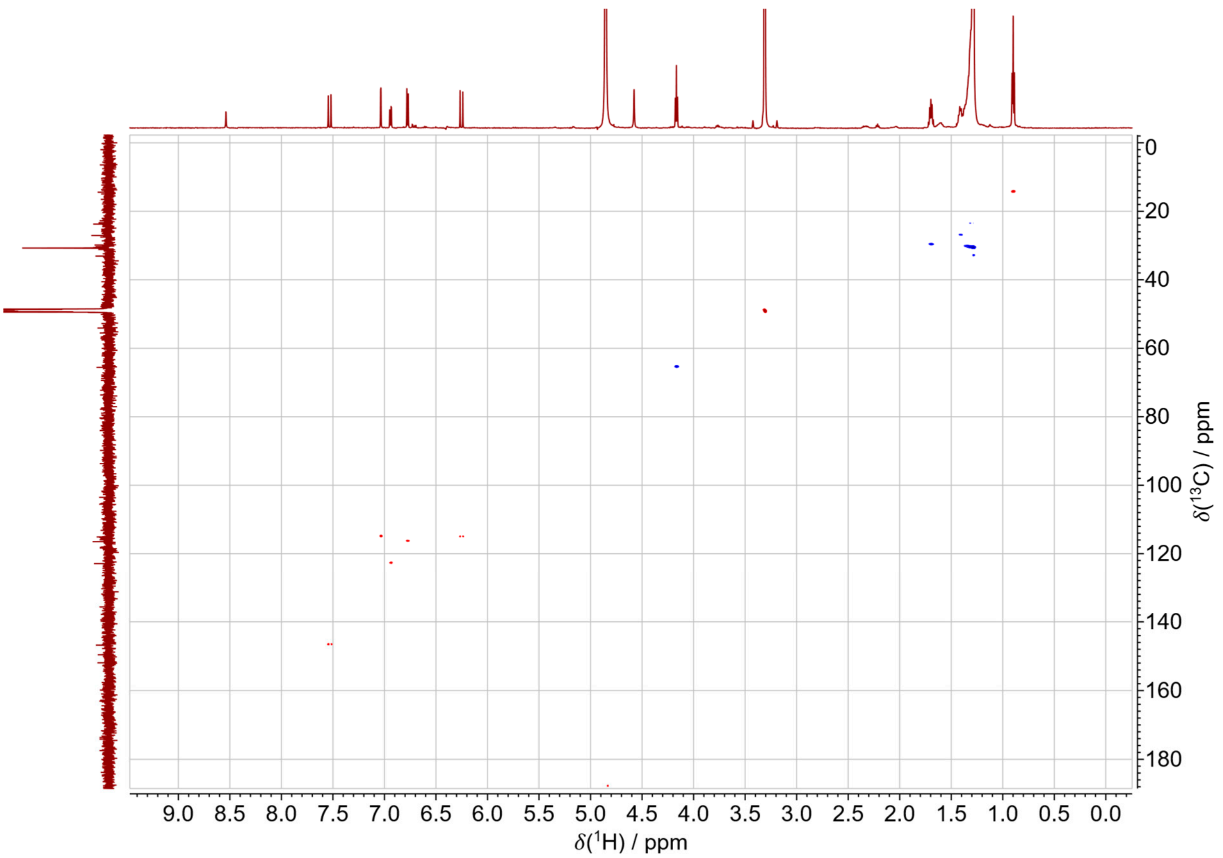

Figure S25. ¹H–¹³C edHSQC NMR spectrum of compound **5** (CD_3OD, 600 and 151 MHz).

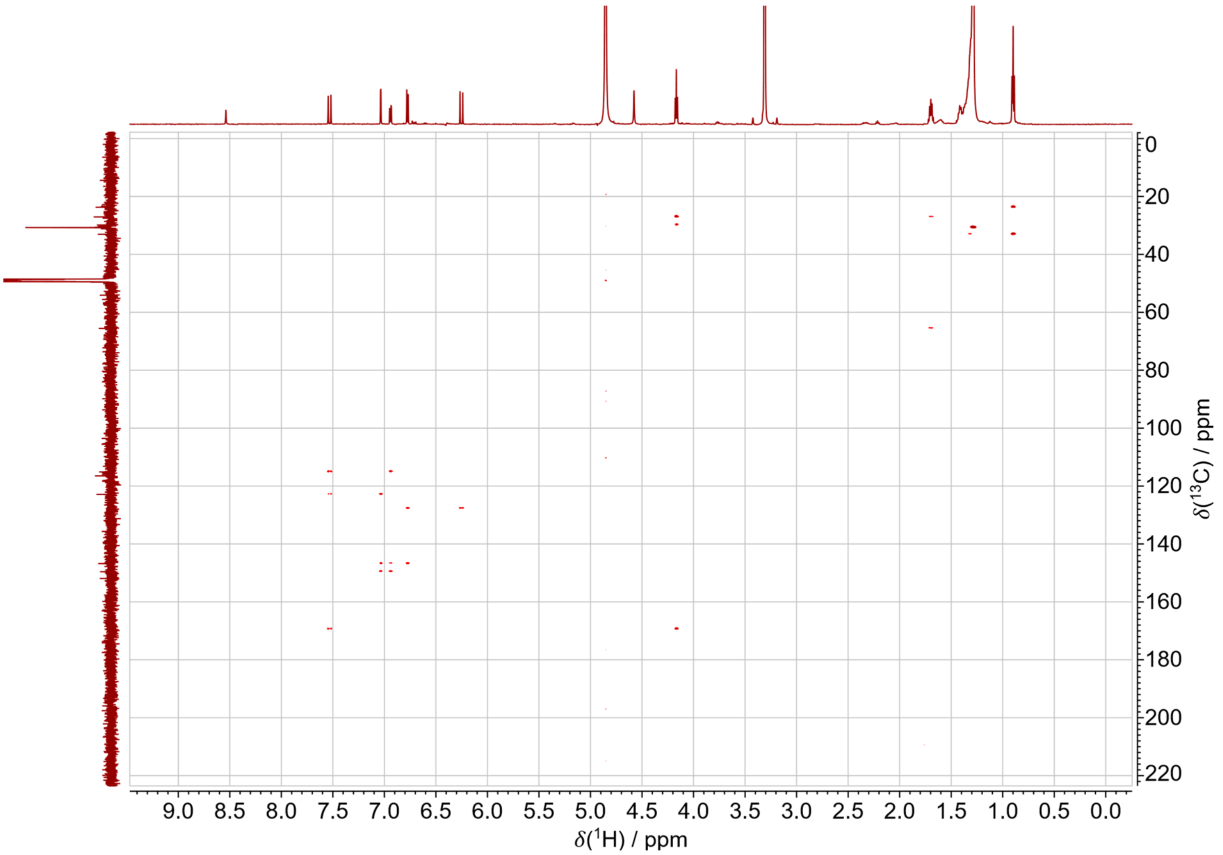

Figure S26. ¹H–¹³C HMBC NMR spectrum of compound **5** (CD_3OD, 600 and 151 MHz).

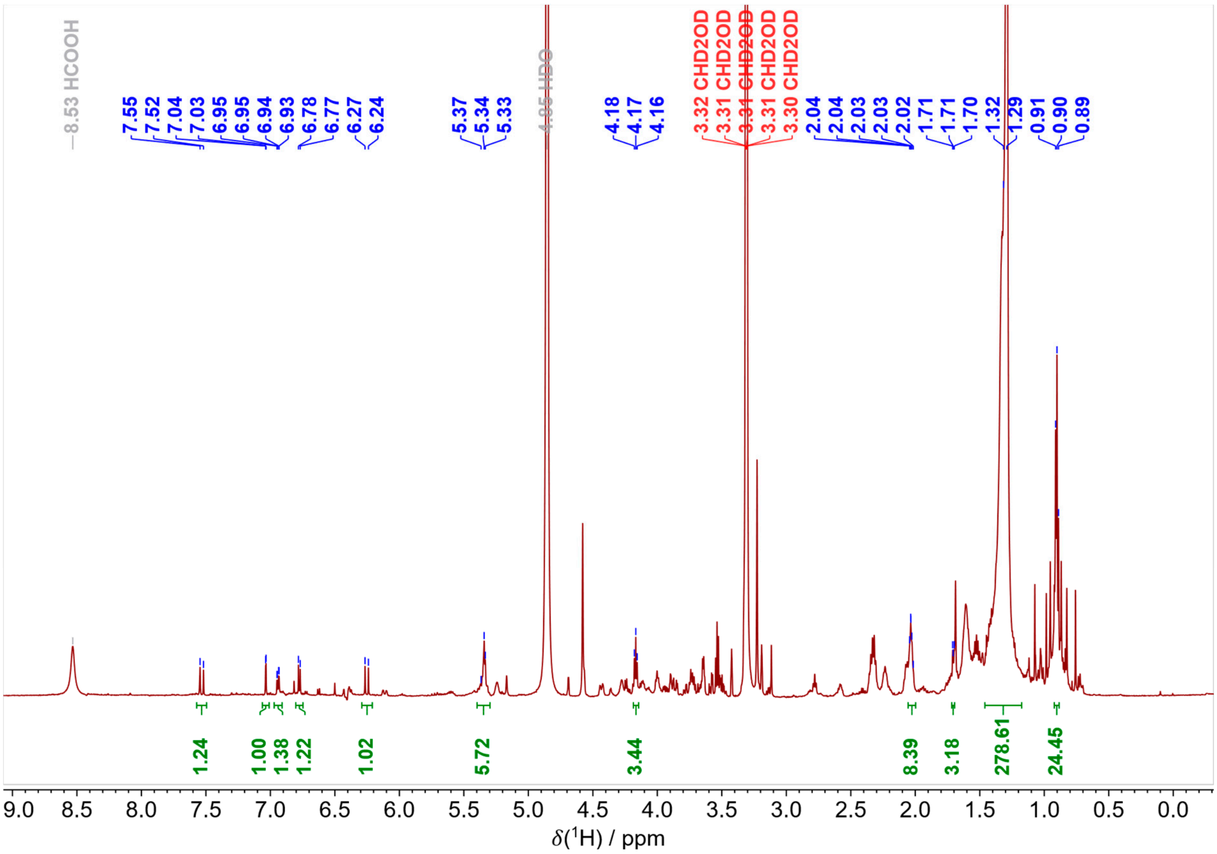

Figure S27. ^{1}H NMR spectrum of compound **6** (CD_3OD, 600 MHz).

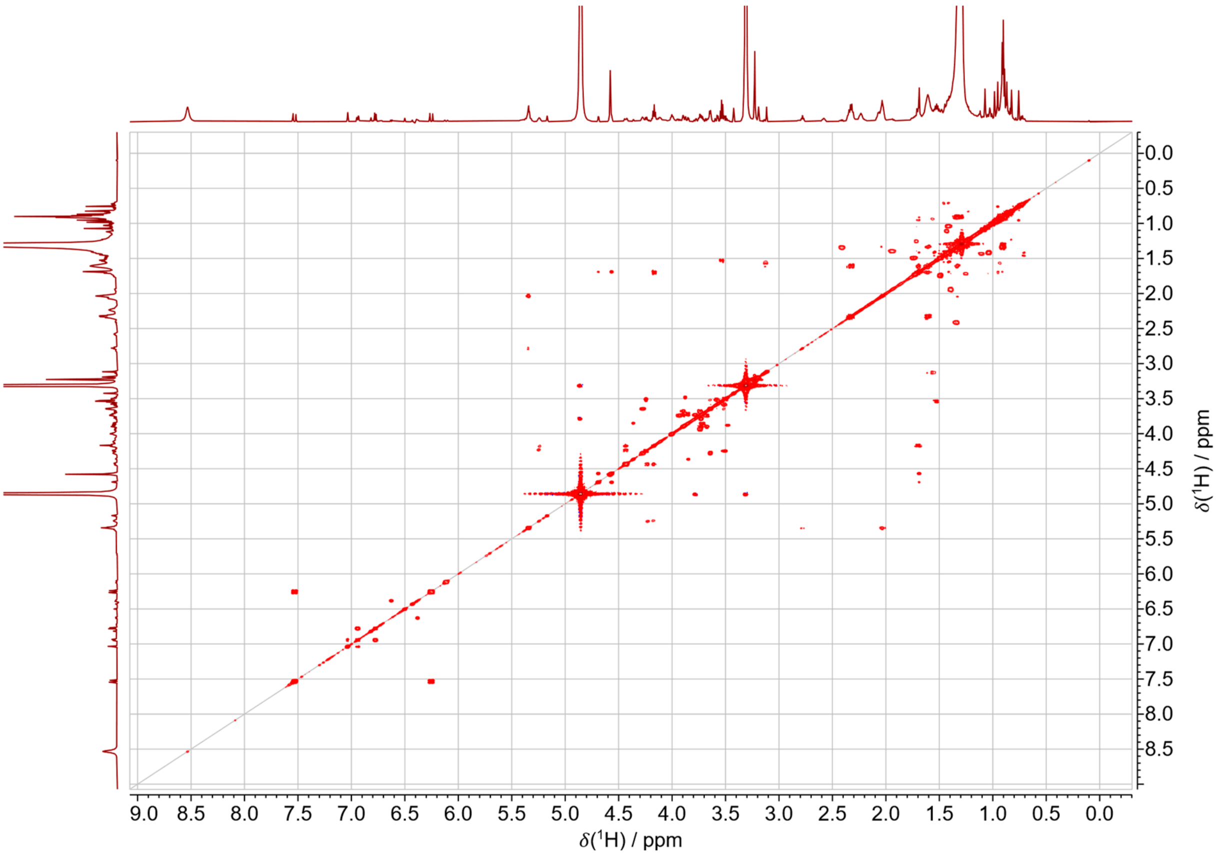

Figure S28. ^{1}H–^{1}H COSY NMR spectrum of compound **6** (CD_3OD, 600 MHz).

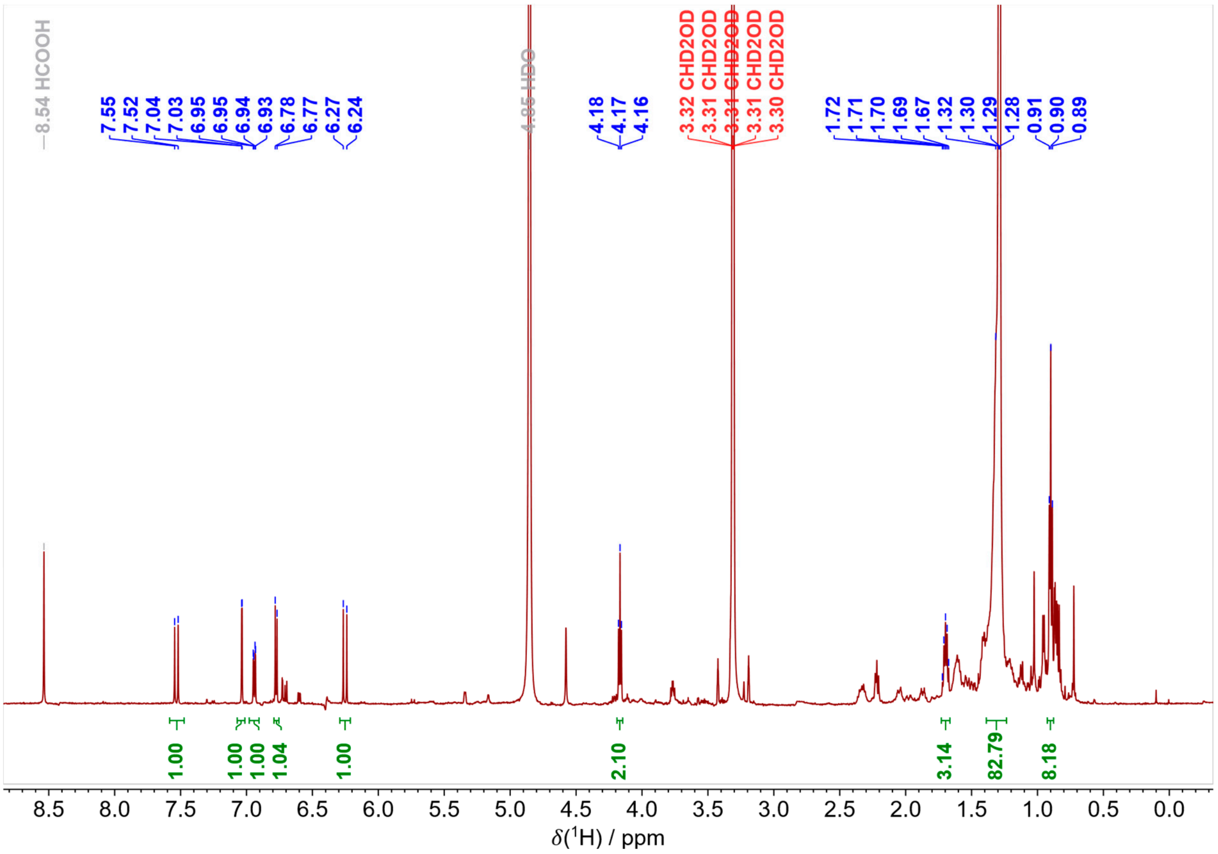

Figure S29. ^{1}H NMR spectrum of compound **7** (CD_3OD, 600 MHz).

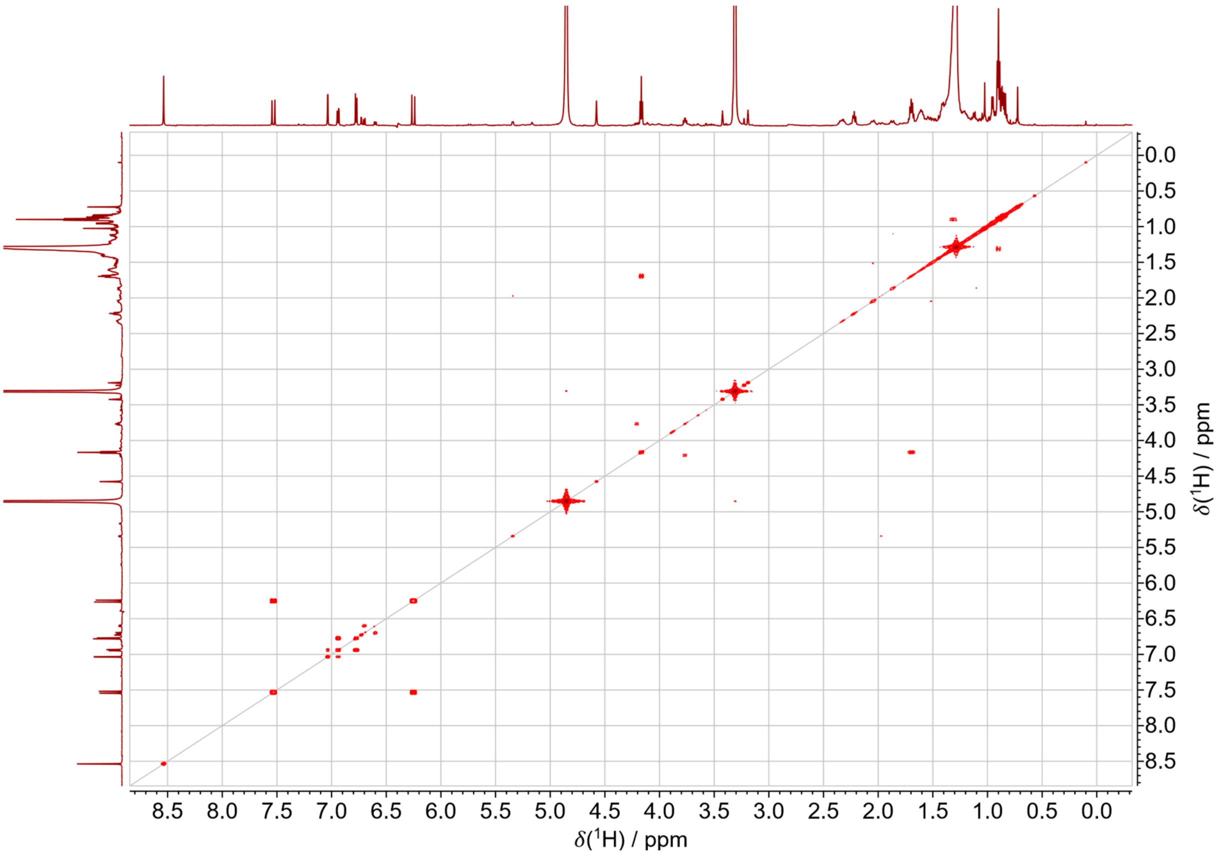

Figure S30. ^{1}H–^{1}H COSY NMR spectrum of compound **7** (CD_3OD, 600 MHz).

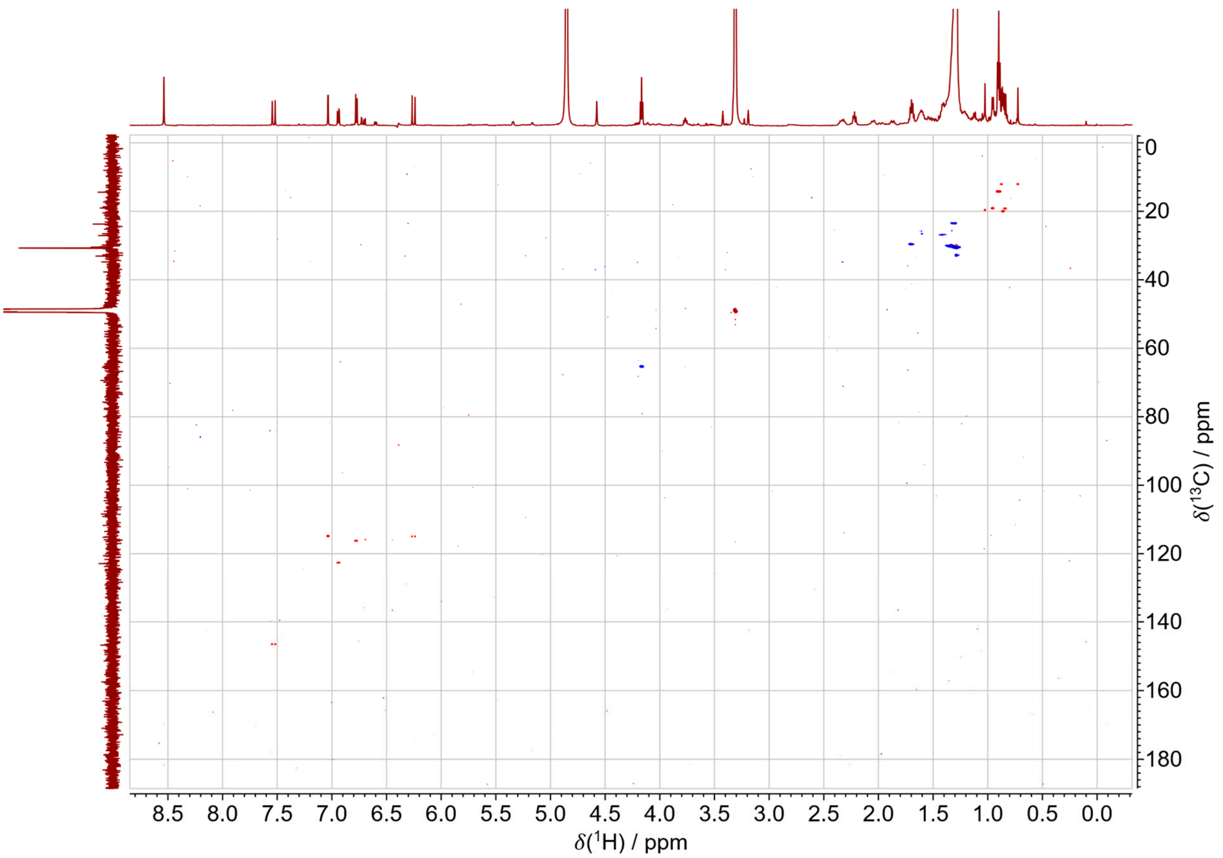

Figure S31. ^{1}H–^{13}C edHSQC NMR spectrum of compound **7** (CD_3OD, 600 and 151 MHz).

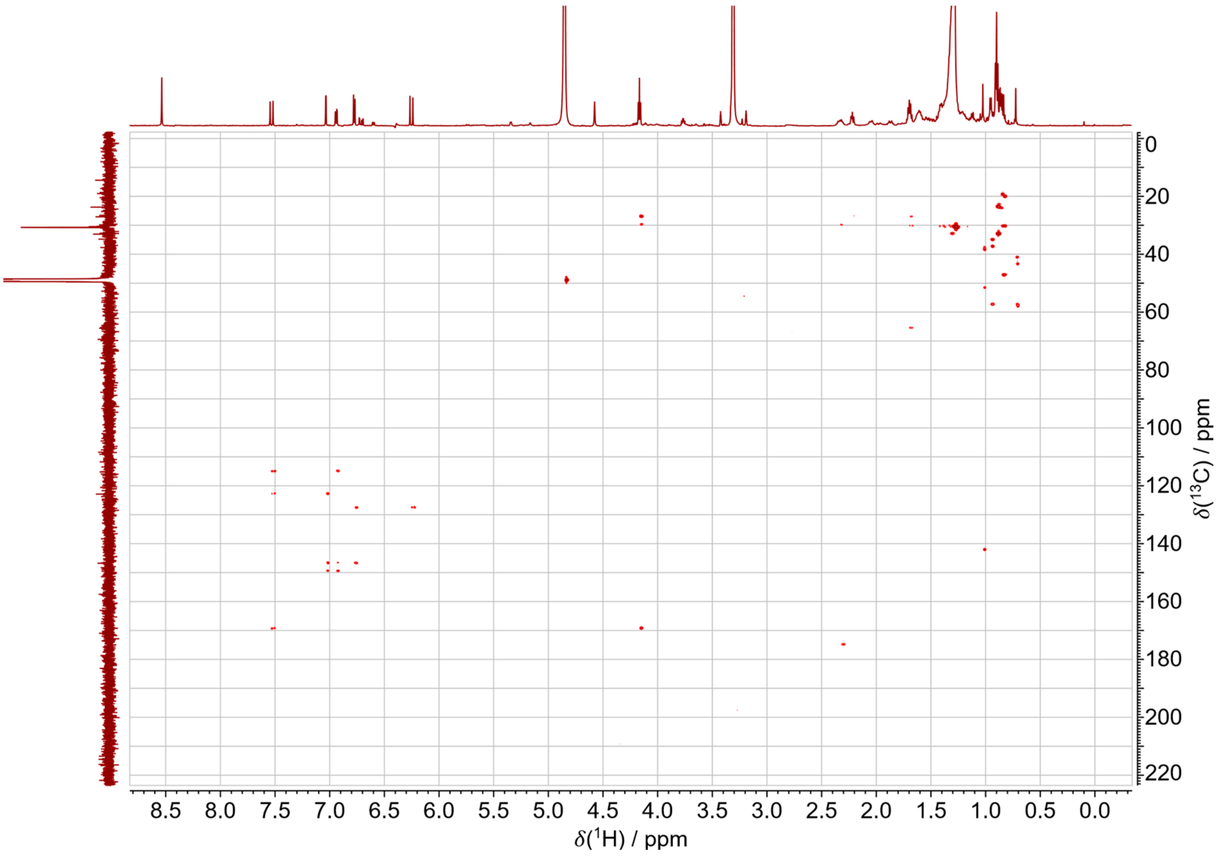

Figure S32. ^{1}H–^{13}C HMBC NMR spectrum of compound **7** (CD_3OD, 600 and 151 MHz).

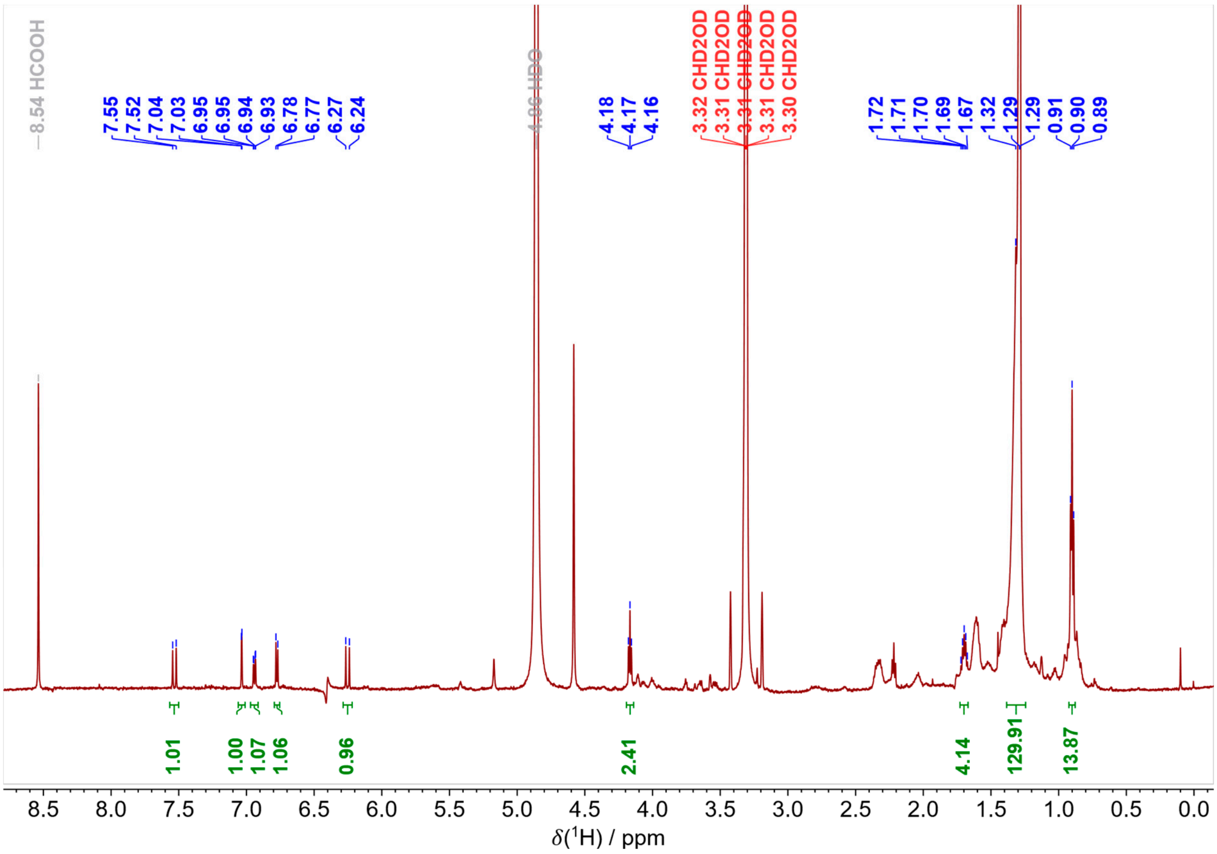

Figure S33. ^{1}H NMR spectrum of compound **8** (CD_3OD, 600 MHz).

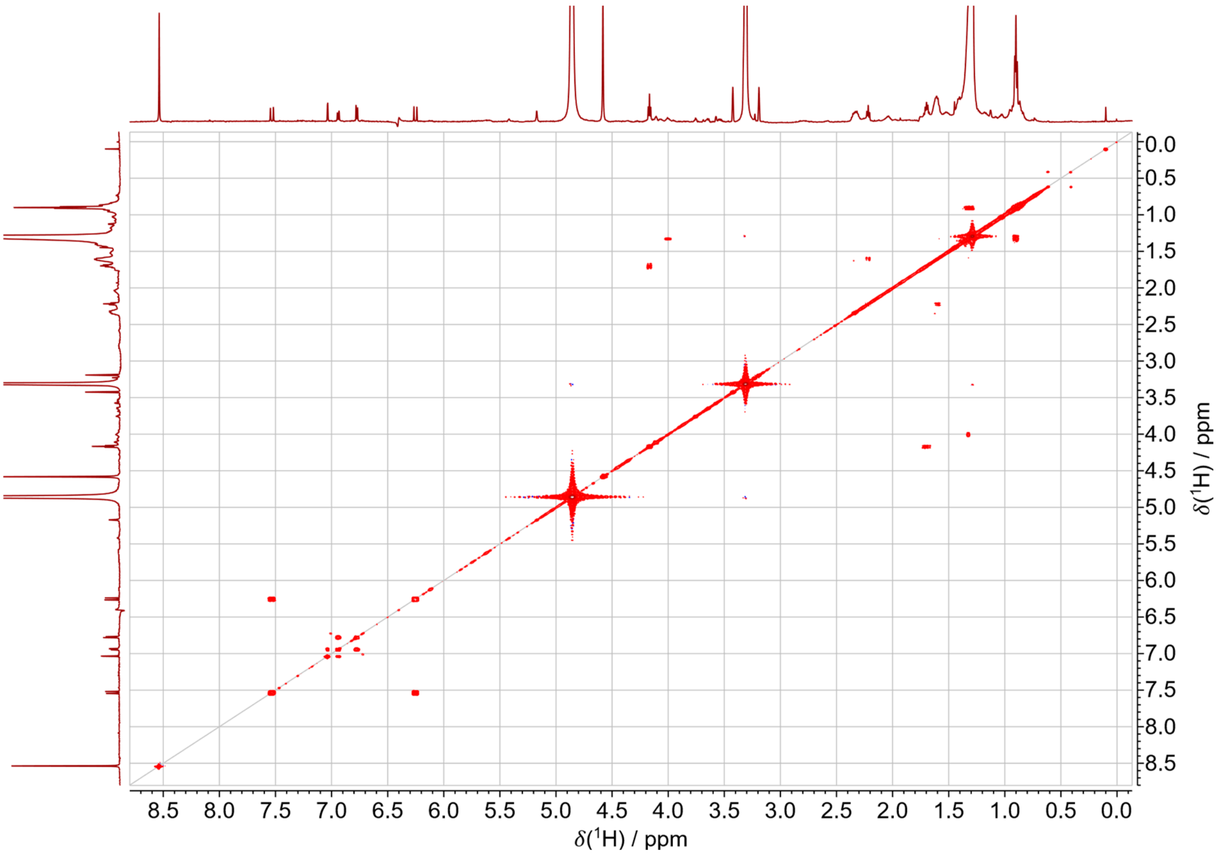

Figure S34. ^{1}H–^{1}H COSY NMR spectrum of compound **8** (CD_3OD, 600 MHz).

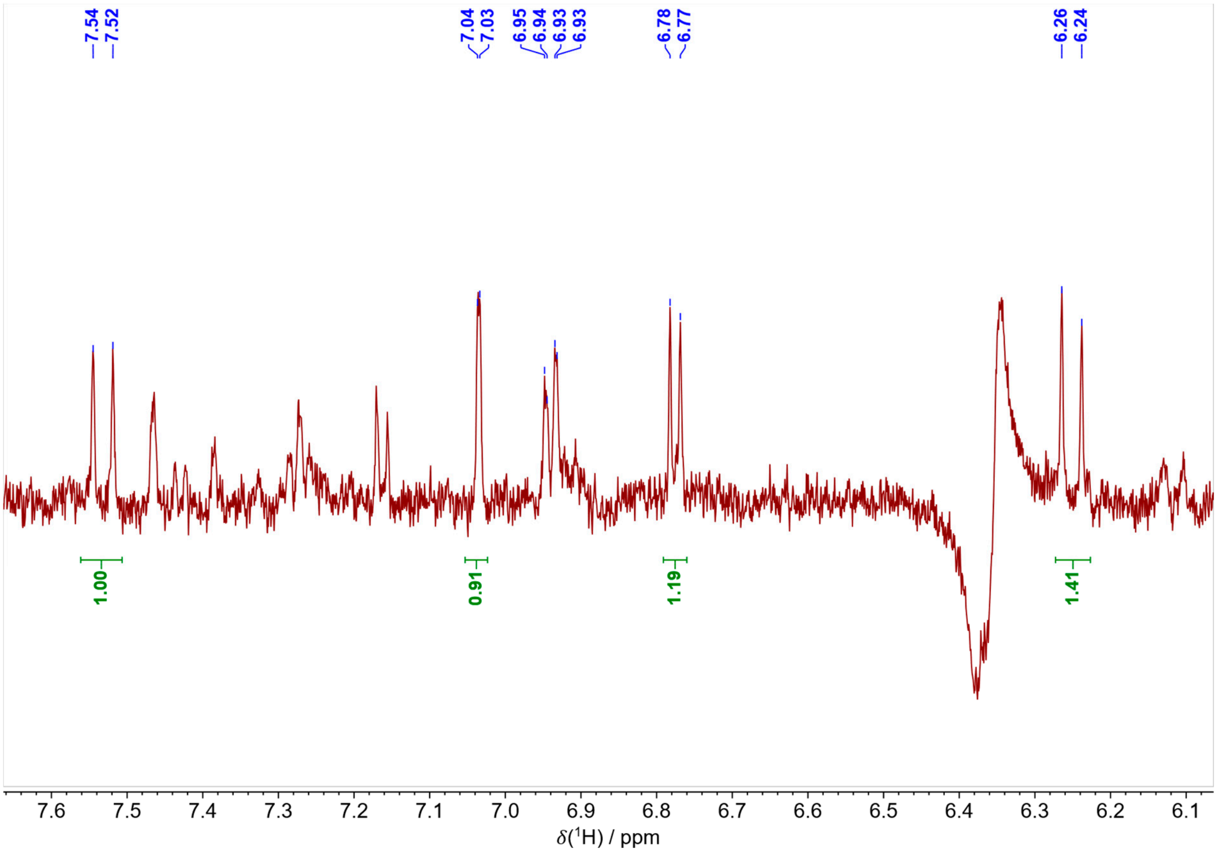

Figure S35. Partial ^{1}H NMR spectrum of compound **9** (CD_3OD, 600 MHz).

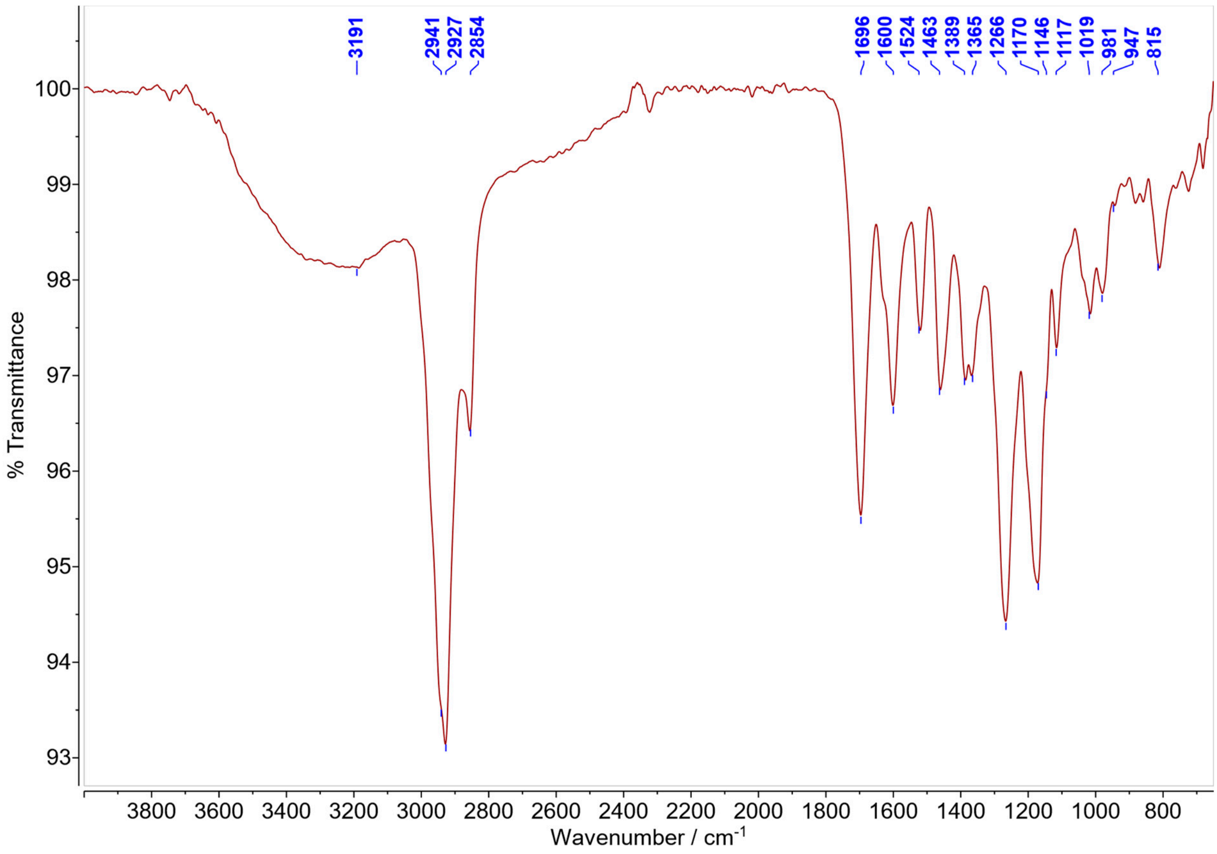

Figure S36. ATR FTIR spectrum of compound **R1**.

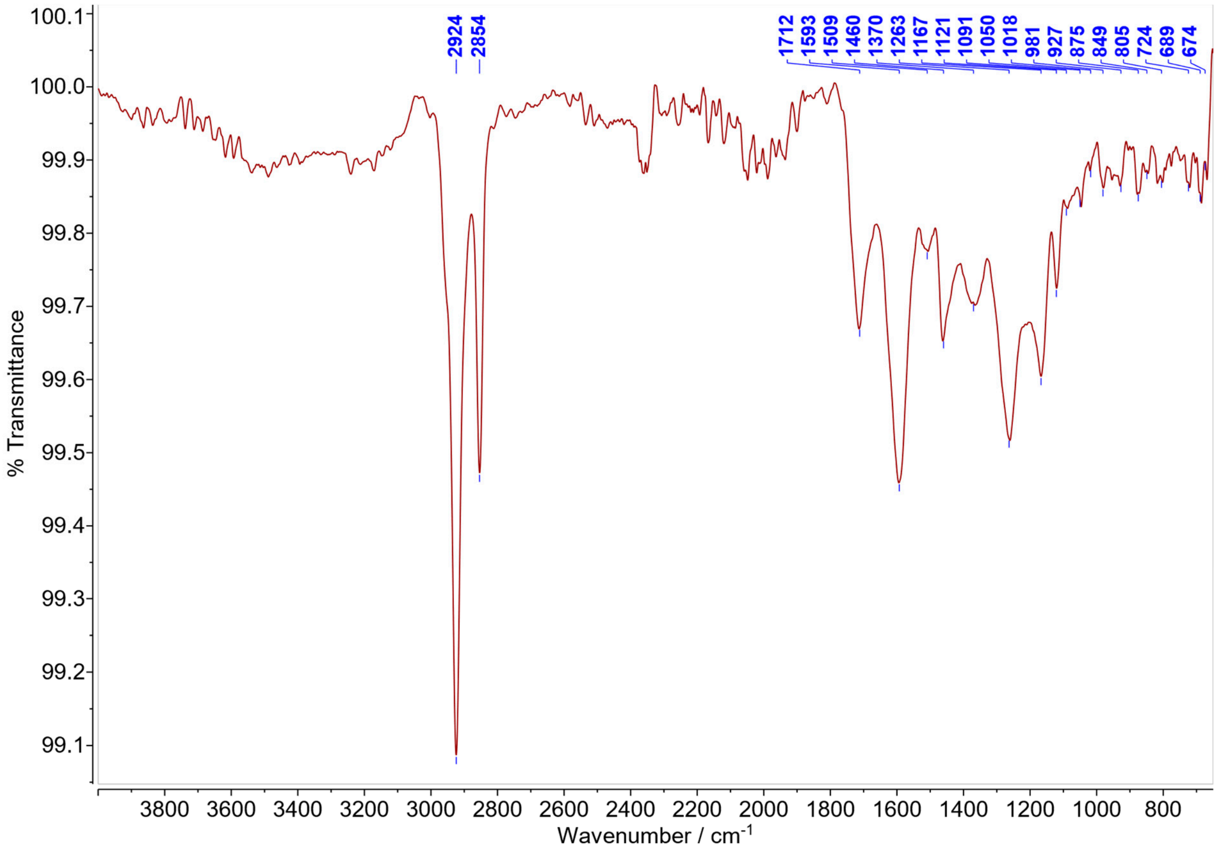

Figure S37. ATR FTIR spectrum of compound **R2**.

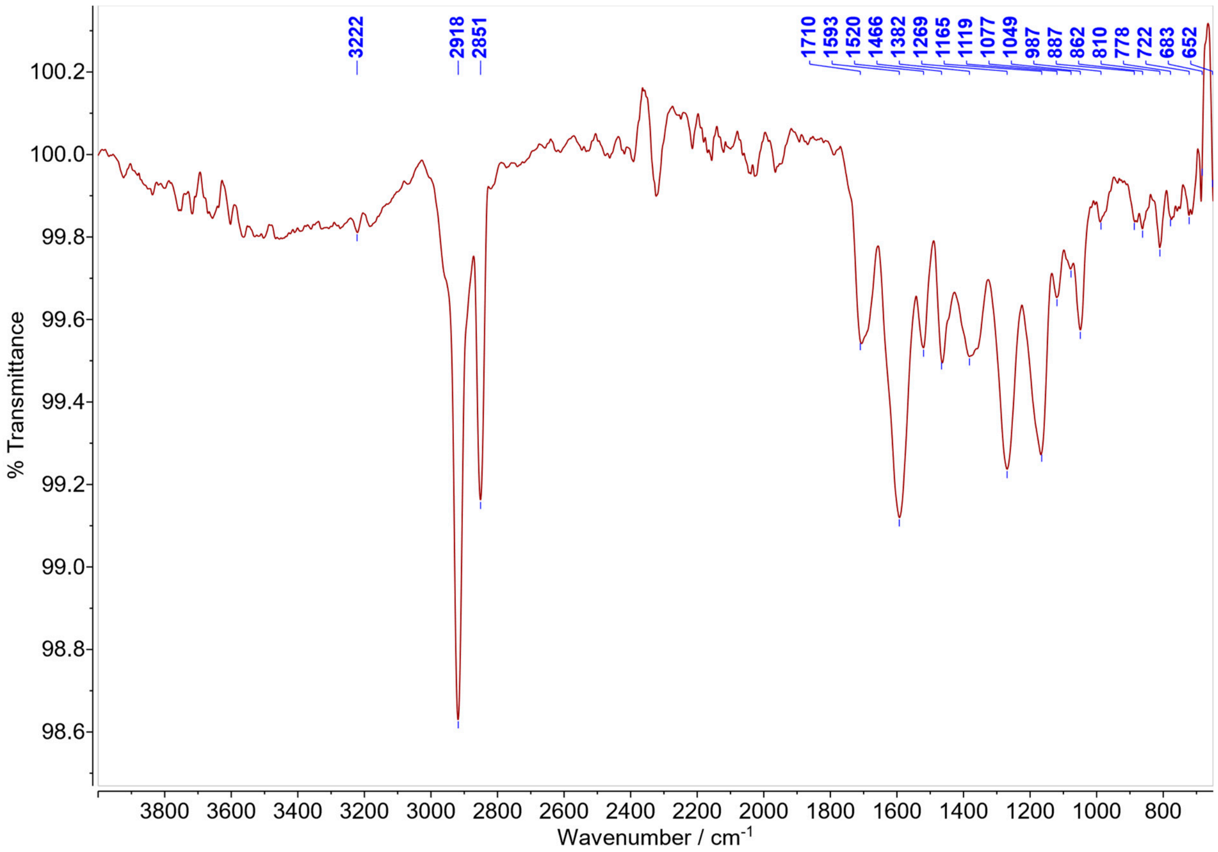

Figure S38. ATR FTIR spectrum of compound **R3**.

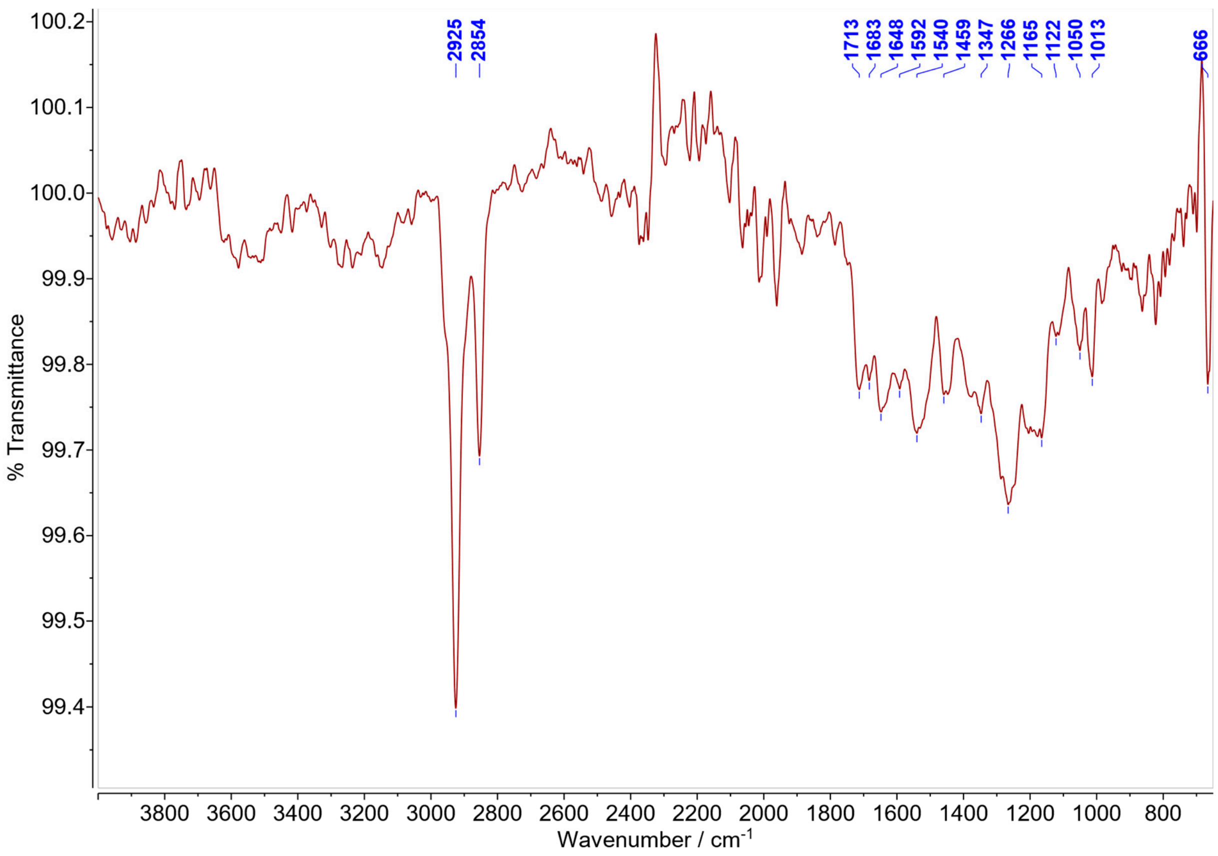

Figure S39. ATR FTIR spectrum of compound **R4**.

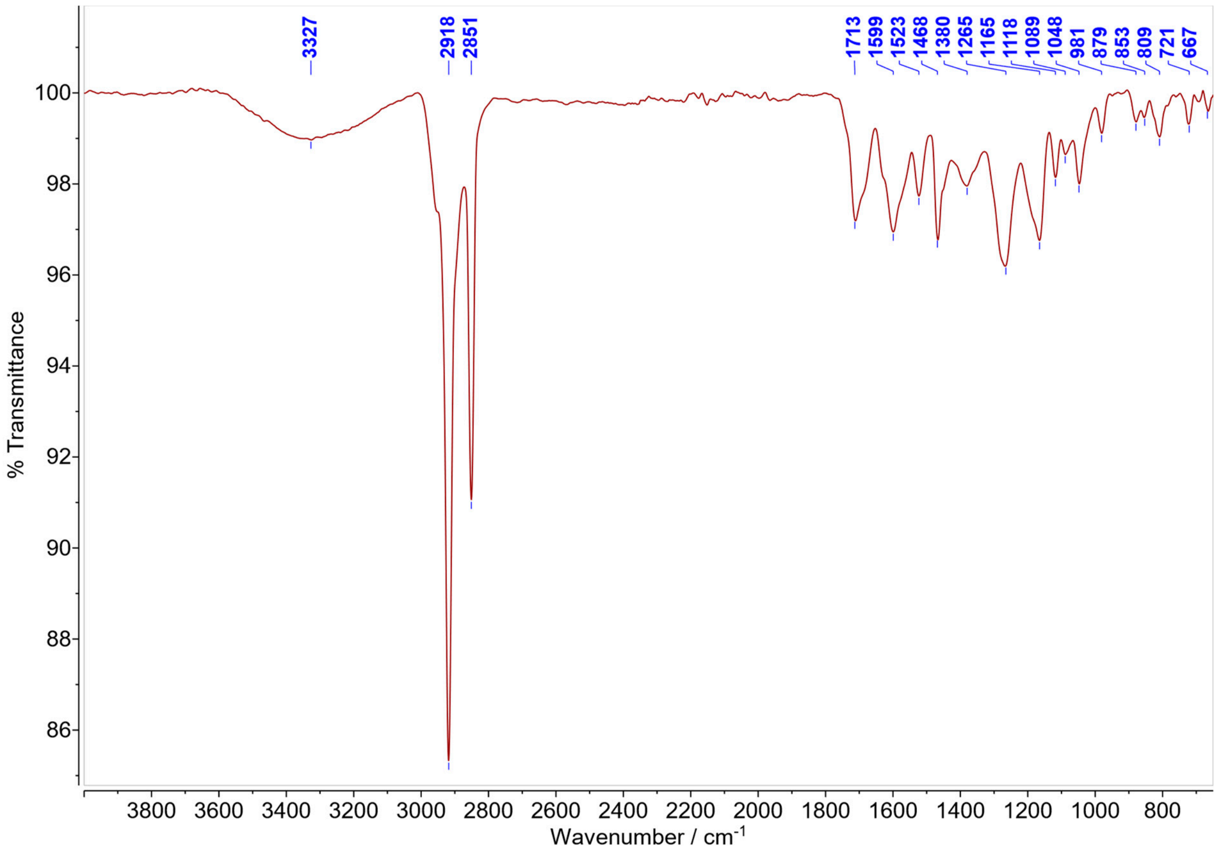

Figure S40. ATR FTIR spectrum of compound **R5**.

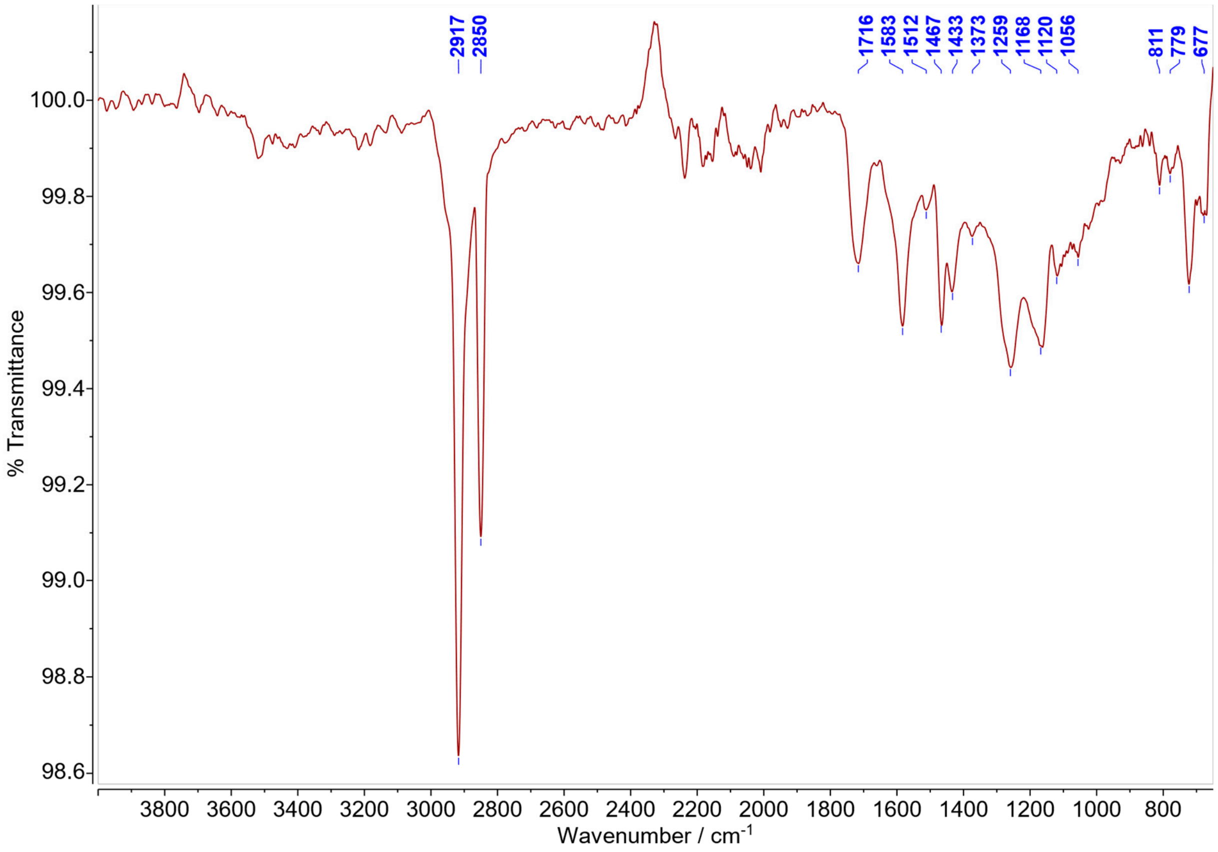

Figure S41. ATR FTIR spectrum of compound **R7**.

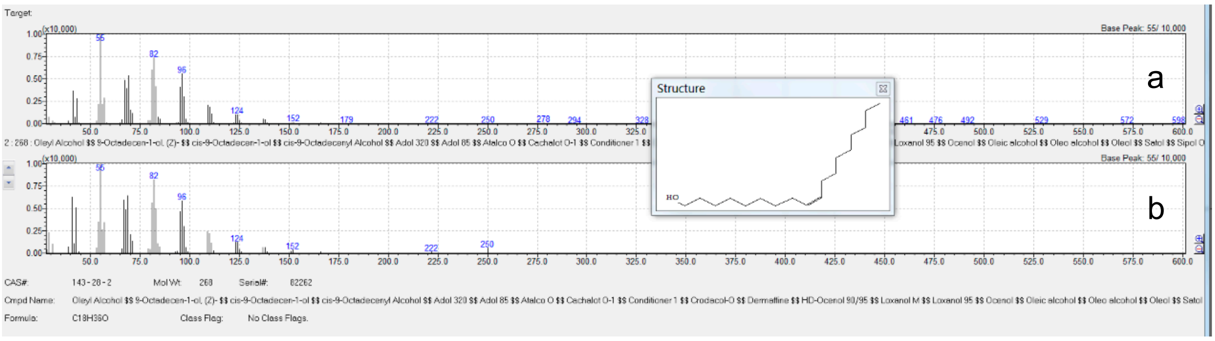

Figure S42. The experimental EI-MS spectrum of compound **R2** (a) and the theoretical EI-MS spectrum of oleyl alcohol (b).

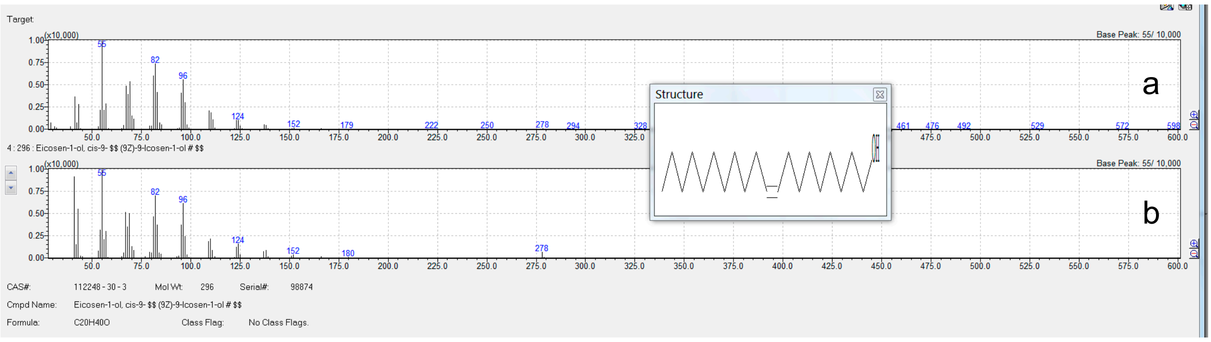

Figure S43. The experimental EI-MS spectrum of compound **R4** (a) and the theoretical EI-MS spectrum of gadoleyl alcohol (b).

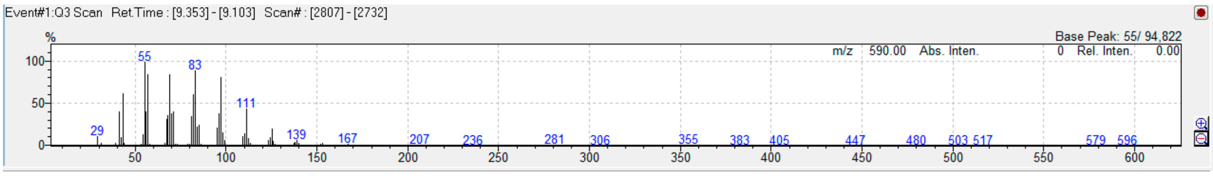

Figure S44. The experimental EI-MS spectrum of compound **R6**.

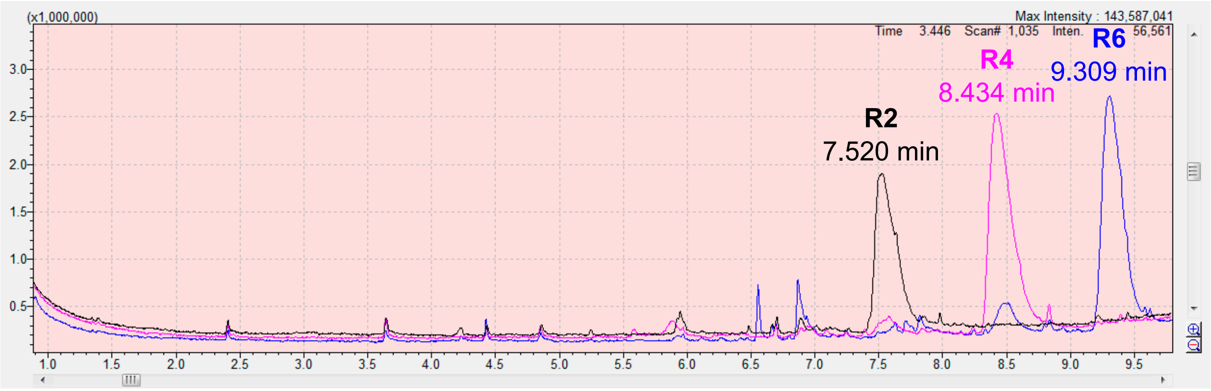

Figure S45. GC–MS TIC chromatograms of compounds **R2** (black), **R4** (magenta), and **R6** (blue). The retention times of **R2**, **R4**, and **R6** are 7.520, 8.434 and 9.309 min, respectively.

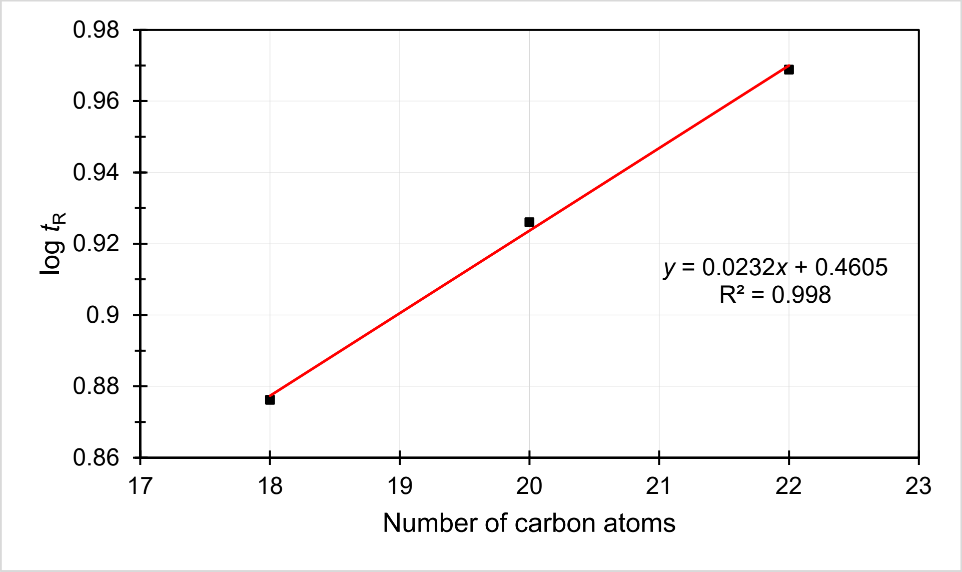

Figure S46. Plot of the logarithmic retention time (t_R) versus the number of carbon atoms for compounds **R2**, **R4**, and **R6** with the fitted line (slope: 0.0232, intercept: 0.4605, R^2 = 0.998).
